# Adjuvant activity of tubeimosides by mediating the local immune microenvironment

**DOI:** 10.3389/fimmu.2023.1108244

**Published:** 2023-02-10

**Authors:** Ziyi Han, Junjie Jin, Xiangfeng Chen, Yanfei He, Hongxiang Sun

**Affiliations:** ^1^ College of Animal Sciences, Zhejiang University, Hangzhou, Zhejiang, China; ^2^ College of Animal Sciences, Wenzhou Vocational College of Science and Technology, Wenzhou, Zhejiang, China; ^3^ College of Food Science and Biotechnology, Zhejiang Gongshang University, Hangzhou, Zhejiang, China

**Keywords:** tubeimosides, adjuvant, structure-activity relationships, network pharmacology, transcriptomics, molecular docking

## Abstract

Rhizoma Bolbostemmatis, the dry tuber of *Bolbostemma paniculatum*, has being used for the treatment of acute mastitis and tumors in traditional Chinese medicine. In this study, tubeimoside (TBM) I, II, and III from this drug were investigated for the adjuvant activities, structure-activity relationships (SAR), and mechanisms of action. Three TBMs significantly boosted the antigen-specific humoral and cellular immune responses and elicited both Th1/Th2 and Tc1/Tc2 responses towards ovalbumin (OVA) in mice. TBM I also remarkably facilitated mRNA and protein expression of various chemokines and cytokines in the local muscle tissues. Flow cytometry revealed that TBM I promoted the recruitment and antigen uptake of immune cells in the injected muscles, and augmented the migration and antigen transport of immune cells to the draining lymph nodes. Gene expression microarray analysis manifested that TBM I modulated immune, chemotaxis, and inflammation-related genes. The integrated analysis of network pharmacology, transcriptomics, and molecular docking predicted that TBM I exerted adjuvant activity by interaction with SYK and LYN. Further investigation verified that SYK-STAT3 signaling axis was involved in the TBM I-induced inflammatory response in the C2C12 cells. Our results for the first time demonstrated that TBMs might be promising vaccine adjuvant candidates and exert the adjuvant activity through mediating the local immune microenvironment. SAR information contributes to developing the semisynthetic saponin derivatives with adjuvant activities.

## Introduction

The development of vaccines has been facing an increasing challenge associated with complicated pathogens such as SARS-CoV-2 (1). Adjuvant is an essential component of the novel vaccines for improving the intensity, nature, breadth, and durability of immune response as well as reducing antigen dose and inoculation times (2). However, very few adjuvants such as aluminum compounds (Alum), AS01, AS03, AS04, CpG ODN, and MF59 have been licensed for clinical use owing to lower potency, severe side effects, and strong toxicity of most of the tested adjuvants (3).

Saponins have been extensively investigated as vaccine adjuvants. Among them, QS-21 possesses the unique capability of eliciting both humoral immune responses and cell-mediated immunity (CMI) including cytotoxic T-lymphocytes (CTLs) (4). Nowadays, QS-21 has been extensively studied for the intracellular pathogen vaccines and cancer vaccines in clinical trials (5, 6). However, QS-21 has inevitable disadvantages including high toxicity, strong hemolysis, low yield, and heterogeneity (7). Indeed, QS-21 is not a single molecule but a 2:1 mixture of two isomeric constituents, QS-21-Api and QS-21-Xyl. It has driven much research for the novel, safe and efficacious saponin adjuvant (8).

Rhizoma Bolbostemmatis, the dry tuber of *Bolbostemma paniculatum* (Maxim.) Franquet (Cucurbitaceae), has being used for treating acute mastitis, inflammatory diseases, tumors, and other diseases in traditional Chinese medicine (9). This crude drug mainly contains saponins, flavonoids, anthraquinones, and alkaloids (10). Among them, triterpenoid saponins, mainly tubeimosides (TBMs), are its major active constituents with various pharmacological actions such as antitumor, anti-inflammatory, and antiviral effects (11–13). TBMs belong to “cyclic bisdesmosides” with two sugar chains at C-3 and C-28 of aglycone linked by 3-hydroxy-3-methyl glutaric acid (14). The adjuvant activities of saponins are close correlated with their chemical structure and are especially affected by two sugar chains at C-3 and C-28 of aglycone and acyl group linked to sugar side chains (15, 16). The chemical structure of cyclic bisdesmosides with a dicrotalic acid bridge could point to adjuvant potentials of TBMs.

The mechanisms of action of saponin-based adjuvants (SBAs), non-targeting pattern recognition receptors, are still poorly understood. The partial knowledge of adjuvants’ mode of action demands more research into their effect on innate immune responses at the site of injection (17). Though different routes of vaccine delivery have been explored in humans, the majority of vaccines are administered intramuscularly. Muscle tissue contains relatively few resident immune cells. Recruitment of immune cells to vaccine delivery sites results in cross-talk between various cells to harmonize innate immune responses, which is critical for interactions between vaccine antigens and immune cells (18). In our previous works, it was found that platycodin D exerted the adjuvant activity through inducing the secretion of cytokines and chemokines at the local tissues *via* activating caspase-1 dependent pyroptosis (19). However, more investigations are needed to uncover the molecular mechanism of SBAs to improve their current application status.

In the present study, three TBMs were isolated and purified from Rhizoma Bolbostemmatis using resin column chromatography and preparative high performance liquid chromatography (HPLC), and then were investigated for their hemolytic activity, toxicity, and adjuvant potentials as well as structure-activity relationship (SAR). Furthermore, the mechanisms of adjuvant action of TBM I were also explored by integrating network pharmacology and transcriptomics and verified using specific signaling pathway inhibitors. We here for the first time reported the adjuvant activity of TBMs and their mechanisms of action.

## Materials and methods

### Materials

3-(4,5-Dimethylthiazol-2-yl)-2,5-diphenyltetrazolium bromide (MTT), concanavalin A (Con A), lipopolysaccharide (LPS), ovalbumin (OVA), type II collagenase, and rabbit anti-mouse horseradish peroxidase (HRP)-IgG were purchased from Sigma-Aldrich, Co., MO, USA; goat anti-mouse HRP-IgG1, IgG2a, and IgG2b were from Southern Biotech. Assoc., AL, USA; RPMI was from Hyclone, Utah, USA; fetal bovine serum (FBS) was from Gibco, Grand Island, NY, USA; DMEM medium was from Corning, NY, USA; mouse ELISA detecting kits were from Wuhan Boster Biological Technology co., Ltd., Hubei, China; TRIzol reagent was from Ambion, Austin, TX, USA; reverse transcriptase, oligo(dT)_18_, and ribonuclease inhibitor were from Shanghai Sangon Biotech Co., Ltd, China; deoxyribonuclease (DNase) I was from Roche Diagnostics, IN, USA; anti-mouse CD3, CD28, CD16/CD32, CD3e–APC, CD4–FITC, CD8–FITC, PE-labeled cytokines, Ly-6G (Gr1)–PE-Cy5, Ly-6C–APC, CD11c–PE, CD3e–PE-Cy5, F4/80–APC, CD117 (c-Kit)–PE-Cy5, and FceR1–APC mAbs were from eBioscience, San Diego, CA, USA; CD45R–PE was from BioLegend, San Diego, CA, USA; Siglec-F–PE was obtained from BD Pharmingen, San Diego, CA, USA; Alexa Fluor 488-conjugated ovalbumin was from Invitrogen, Carlsbad, CA, USA; bicinchoninic acid (BCA) protein assay kit, enhanced chemiluminescence (ECL) kit, HRP-conjugated goat anti-rabbit and anti-mouse IgG (H+L), and radioimmunoprecipitation assay (RIPA) lysis buffer were acquired from Beyotime Biotech, Nantong, Jiangsu, China; SYK inhibitor R406, LYN inhibitor SU6656, and STAT3 inhibitor S3I-201 were from Selleck Chemicals, Houston, TX, USA; FastStart universal SYBR Green Master, the phosphatase inhibitor cocktail and protease inhibitor cocktail were acquired from Bimake, Houston, TX, USA; rabbit anti-mouse SYK, STAT3, phospho-SYK, and phospho-STAT3 mAbs were from Cell Signaling Technology, Danvers, MA, USA; anti-mouse TATA-box binding protein (TBP) mAb was from Proteintech, Chicago, IL, USA; blue plus IV protein marker (DM131) was from TransGen Biotech, Beijing, China. The SurePrint G3 8 × 60 K mouse gene expression microarray was provided by Agilent Technologies, Santa Clara, CA, USA.

### Extraction, isolation and identification of TBMs

Rhizoma Bolbostemmatis was purchased from Hangzhou Huqing Yutang Pharmaceutical Co., Ltd (Zhejiang, China) and was authenticated as the dry tuber of *B. paniculatum*. The drug (1 kg) was powdered and then extracted with 70% ethanol under reflux twice. After filtration through 3MM *Whatman* filter paper (thickness: 0.34 mm), excess solvent was recovered under reduced pressure. The ethanol extract was subjected to D101 resin washed with water and sequentially eluted with 30%, 50%, and 70% ethanol as elution to obtain three fractions. The yields of three fractions were 0.82%, 2.62%, and 0.53% of the dried crude drug (*w*/*w*) for BPS30, BPS50, and BPS70, respectively. BPS50 (1.0 g) was dissolved in 5 mL of 50% ethanol and then passed through a 0.45-μM filter. The solution was applied to SinoChrom ODS-BP column (250 mm × 20 mm, 10 μm) using 43% ethanol as mobile phase and UV230-II UV-Vis detector on Elite P270II preparative HPLC instrument (Dalian, China) to give TBM I (510.7 mg), TBM II (98.9 mg), and TBM III (190.4 mg).

**Figure 1:**
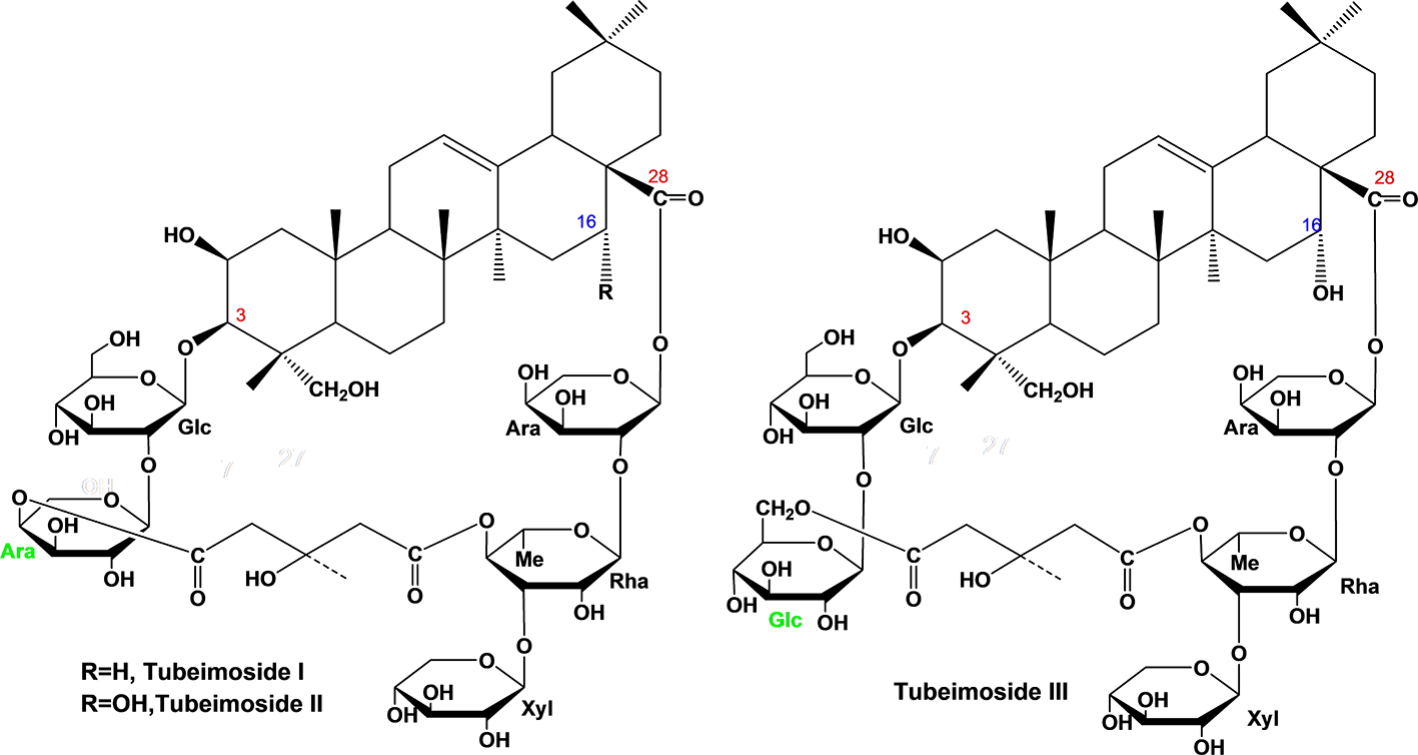
Chemical structure of three tubeimosides isolated from Rhizoma Bolbostemmatis. The chemical structures were identified by HR-ESI-MS and NMR. Ara, *α*-L-arabinopyranosyl; Glc, *β*-D-glucopyranosyl; Rha, *α*-L-rhamnopyranose; Xyl, *β*-D-xylopyranosyl.

Each compound was identified by high resolution electrospray ionization mass spectroscopy (HR-ESI-MS) on OrbitrapElite Mass spectrometer (Thermo Scientific, Bremen, Germany) and nuclear magnetic resonance spectroscopy (NMR) on Agilent DD2-600 NMR spectrometer. The quasi-molecular ions at *m/z* 1317.6167, 1333.6135, and 1363.6233 ([M–H]^–^) were in agreement with the molecular formula C_63_H_98_O_29_ (calc. 1318.6174), C_63_H_98_O_30_ (calc. 1334.6143), and C_64_H_100_O_31_ (calc. 1364.6249) for TBM I, TBM II, and TBM III, respectively (**Figure 1**). Their NMR data were listed in **Table S1**.

### Experimental animals

sICR and C57BL/6J mice aged 5–6 weeks were purchased from Shanghai SLAC Laboratory Animal Co., Ltd, China. All experiments complied with China legislation on laboratory animals and were authorized by the Experimental Animal Ethics Committee of Zhejiang University.

### Haemolytic assay

The hemolytic activity of TBMs and Quil A was examined using 0.5% rabbit red blood cell (RBC), and the results were expressed by the concentration resulting in 50% of the maximum hemolysis (HD_50_) using NDST software (20).

### Toxicity observation

ICR mice were divided into six groups with 5 mice per group. Animals were subcutaneously (*s.c.*) injected with TBM I, TBM II, and TBM III at a single dose of 1.0 mg or Quil A (150 and 200 μg) in 0.2 mL saline, and then monitored for body weight, swelling and loss of hair at the injection site, and lethality daily for 1 week (21).

### Immunization

Mice were divided into 12 groups with 5 mice per group. Mice were *s.c.* injected with OVA alone (25 μg) or in combination with TBMs (25, 50, or 100 μg) or Quil A (10 μg) in 200 μL PBS. An intensive immunization was given 14 days later. Animals injected with PBS served as the control. Splenocytes and serum were prepared 2 weeks after the second inoculation.

### Injections

C57BL/6J mice were intramuscularly (*i.m.*) injected in the quadriceps tissues on one leg with 50 μg TBM I dissolved in 50 µL of PBS and on the contralateral leg with 50 μL PBS as control. Mice were sacrificed at the indicated time points, and then the quadriceps muscle tissues were taken from four mice per group. For enzyme-linked immunosorbent assay (ELISA), the muscle tissues were homogenized using Tissue Ruptor Disposable probes (QIAGEN, Germany) in 1 mL PBS with protease inhibitor cocktail. After centrifugation at 12000 rpm for 10 min at 4 °C, the protein concentrations in the supernatants were detected by the BCA assay kit. For the real-time quantitative PCR (RT-qPCR), the muscle tissues were homogenized with 1 mL TRIzol reagent using Tissue Ruptor Disposable probes (19).

### Serum antigen-specific antibody measurement

Serum antigen-specific IgG antibody and its isotype titers in OVA-immunized mice were determined by an indirect ELISA (20).

### Splenocyte proliferation assay

Splenocytes (5 × 10^5^ cells/well) were seeded into 96-well cell culture plates, and then incubated with Con A (5 μg/mL), LPS (10 μg/mL), OVA (20 μg/mL), or RPMI for 48 h at 37°C and 5% CO_2_. Four hours before the end, the cell proliferation was detected using MTT assay (20).

### Natural killer cell activity assay

Splenocytes (1 × 10^6^ cells/well) and human leukemia K562 cells (2 × 10^4^ cells/well) were seeded in 96-well U-bottom microtiter plates in RPMI complete medium and then incubated for 20 h at 37°C in 5% CO_2_. The killing activities of NK cells in splenocytes against K562 cells were assured by MTT assay (20).

### Cytokine and chemokine analysis

Splenocytes (5 × 10^6^ cells/well) were stimulated with OVA (20 μg/mL) in 24-well cell culture plates for 72 h at 37°C and 5% CO_2_. The levels of cytokines and chemokines in the splenocyte culture supernatants and mouse muscle tissue homogenates were detected using commercial ELISA kits as previously described (19). For quadriceps muscle tissues, the values were expressed as pg/mg protein.

### RT-qPCR

Splenocytes (5 × 10^6^ cells/well) were stimulated with OVA (20 μg/mL) in 24-well cell culture plates for 18 h at 37°C and 5% CO_2_. The total RNA was isolated with TRIzol reagent and reverse transcription was performed. The PCR was conducted on a Bio-Rad CFX 96 system with specific primers (**Table S2**) using SYBR Green qPCR Master Mix (19). The expression levels of the tested genes relative to GAPDH were determined using the 2^-ΔΔCt^ method.

### Delayed type hypersensitivity

ICR mice were *s.c.* injected at the left hind limbs with OVA alone or in combination with TBMs (25, 50, or 100 μg) or Quil A (10 μg) for sensitization. Five days later, two hind footpads were *s.c.* injected with and without OVA (10 μg) in 50 μL PBS, respectively. The thickness of footpad was measured using a vernier caliper 24 h after the challenge. The difference value in left and right footpad thickness was represented as the footpad swelling (20).

### Intracellular cytokine assay

Splenocytes (5 × 10^5^ cells/well) were incubated in 96-well cell culture plates with OVA (20 μg/mL) and anti-CD28 antibody (5 μg/mL) for 12 h at 37°C in 5% CO_2_. Cells incubated with anti-CD28 alone or in combination with anti-CD3 antibody served as the negative and positive control, respectively. Brefeldin A was added to each well for an extra 4 h. The pelleted cells were blocked with anti-mouse CD16/CD32 antibody on ice for 10 min. Followed by washing with 5% FCS-PBS, CD3e–APC and CD8α–FITC or CD3e–APC and CD4–FITC were added, and the mixture was incubated for 15 min at 25°C. After washing, cells were fixed with IC fixation buffer at 25°C for 30 min in the darkness. Permeabilization buffer and anti-mouse IL-2–PE, IL-4–PE, IL-10–PE, or IFN-γ–PE were added, and incubated at 25°C for 30 min in dark (22). The stained cells were analyzed on BD FACSCanto II flow cytometer (BD Biosciences) using FlowJo vX.0.7 (Tree Star).

### Flow cytometry for immune cells

C57BL/6J mice were injected *i.m.* with 25 μL/quadriceps muscle of OVA-AF488 (10 μg) alone or in combination with TBM I (25, 50, or 100 μg). Quadriceps muscles and draining lymph nodes (dLNs) were harvested from four mice per group at 12, 24, and 48 h after administration, respectively. Quadriceps muscles were cut into small pieces and digested with 100 µg/mL DNaseI, 0.05% type II collagenase, and 1% FCS in DMEM for 30 min at 37 °C. After centrifugation, the pelleted cells were resuspended in DMEM and then filtered through a 70 μm nylon mesh to obtain a cell suspension. Cell suspension was centrifuged and washed with PAB (1% FBS in PBS). The dLNs were homogenized within PBS using glass homogenizers gently. After centrifugation, the pelleted cells were washed with PAB. The cells were blocked with 1 μg of purified anti-mouse CD16/CD32 antibody for 10 min to inhibit non-specific staining, and then stained with combinations of anti-mouse Ly-6G–PE-Cy5, Ly-6C–APC and CD11c–PE, or CD3e–PE-Cy5, F4/80–APC and CD45R–PE, or CD117–PE-Cy5, FceR1–APC and Siglec-F–PE at room temperature for 30 min (19). The stained cells were analyzed using a FACSCanto II Flow cytometer. Data analysis was performed with FlowJo vX.0.7.

### Microarray analysis

The quadriceps muscles harvested from three mice per group at 4 h after injection were subjected to transcriptomic analysis using Agilent SurePrint G3 mouse gene expression microarray as previously described (19). The microarray data set (GSE205650) has been deposited in the GEO database (https://www.ncbi.nlm.nih.gov/geo/query/acc.cgi?acc=GSE205650). Differentially expressed genes (DEGs) were selected by *P*-value<0.05 and fold change >2. The Gene Ontology (GO) and Kyoto Encyclopedia of Genes and Genomes (KEGG) enrichment analyses were performed using Metascape (http://metascape.org/gp/index.html#/main/step1) (23).

### Gene set enrichment analysis

The whole genes detected in the quadriceps muscles were subjected to GSEA Software (version 4.2.1) using GSKB database. Leading-edge subsets (LESS) were screened based on |Normalized enrichment score (NES)| > 1, *P* < 0.05, and false discovery rate (FDR) < 0.25. Multiple GSEA plots were produced by ‘plyr’, ‘ggplot2’ and ‘grid’ packages (24). The genes in each LESS were integrated as core genes. The protein-protein interaction (PPI) network of core genes was constructed using STRING database version 11.5 (https://cn.string-db.org/). The hub genes were identified by using the cytoHubba plug-in of Cytoscape with seven common algorithms (MCC, MNC, Degree, Closeness, Radiality, Stress, and EPC). TRRUST database (https://www.grnpedia.org/trrust/) was used to predict the upstream transcription factors (TFs) of the core genes, and an adjusted *P* < 0.05 was considered significant (25).

### Network pharmacological analysis

PubChem (https://pubchem.ncbi.nlm.nih.gov/), Swiss target prediction (http://www.swisstargetprediction.ch/), and SuperPred database (https://prediction.charite.de/s) were used to predict the potential targets of TBM I. The common genes between the predicted targets and core genes from GSEA were considered as candidate targets of TBM I. The PPI network was constructed using STRING database version 11.5 (https://cn.string-db.org/). The plug-in molecular complex detection technology (MCODE) in Cytoscape 3.9.1 was used to analyze pivotal functional modules by the following criteria: K-core = 2, degree cutoff = 2, max depth = 100, and node score cutoff = 0.2. Subsequently, a co-expression network of these key targets involved in the module was constructed using GeneMANIA (http://www.genemania.org/) (26).

### Molecular docking

The molecular docking simulation was performed using AutoDockTools (4.2.3) to verify the credibility of the hub targets as previously described (27). The mechanical structure of TBM I was optimized *via* Chem3D (21.0). The 3D structures of the proteins were obtained from the PDB database (http://www.rcsb.org/pdb/home/home.do). The ligand-receptor binding property was analyzed. Binding energies < -1.2 kcal/mol denoted the feasible docking, and the results were visualized using Pymol software.

### Cell culture and stimulation

The mouse C2C12 myoblast cell line was purchased from the cell bank of the Shanghai Branch of the Chinese Academy of Sciences and maintained in a 5% CO_2_ atmosphere in DMEM complete medium supplemented with 10% FBS, 100 µg/mL streptomycin, and 100 U/mL penicillin. C2C12 cells were plated 24 h before stimulation and were incubated with TBM I at various concentrations for the indicated times. Then, the cells and culture supernatants were collected for detecting cell proliferation, mRNA and protein expression levels using MTT assay, ELISA, RT-qPCR, and Western blotting, respectively.

### Inhibition assay

After pretreatment with R406 (SYK inhibitor, 8 µM), SU6656 (LYN inhibitor, 2 µM), or S3I-201 (STAT3 inhibitor, 100 µM) for 1 h, C2C12 cells were stimulated with TBM I (40 µM) for 4 h, 8 h, or 12 h. The cells and supernatants were collected for the mRNA and protein expression levels by RT-qPCR, ELISA, and Western blotting.

### Statistical analysis

All results were presented as mean ± SD and subjected to ANOVA and Student’s *t*-tests for calculating the statistical significance of difference, and *P* < 0.05 was identified as statistically significant.

## Results

### Hemolytic activity and toxicity of TBMs

The levels of endotoxin in the solution of three TBMs (10 mg/mL) were determined to be lower than 0.5 endotoxin units (EU)/mL using tachypleus amebocyte lysate assay. Therefore, these samples were all excluded from endotoxin contamination.

The HD_50_ values of TBM I, TBM II, TBM III, and Quil A were 5.39 ± 0.06, 7.91 ± 0.22, 4.48 ± 0.11, and 4.60 ± 0.02 μg/mL against 0.5% rabbit RBC, respectively. Among three TBMs, the hemolytic activity with the following order: TBM III > TBM I > TBM II.

When the animals were administered with TBMs at a single dose of 1.0 mg per mouse, there is no lethality observed except 2 mice died of TBM II during the monitoring period. Local swelling and loss of weight were found in the mice treated with TBM II and TBM III within 72 h after injection. Under the same condition, the local swelling, loss of hair, and loss of weight occurred in the Quil A-injected mice. Moreover, the numbers of deaths in five mice injected with Quil A were 2 and 3 for the doses of 150 and 200 μg per mouse, respectively.

### TBMs potentiated humoral and cellular immune responses to OVA in mice

**Figure 2:**
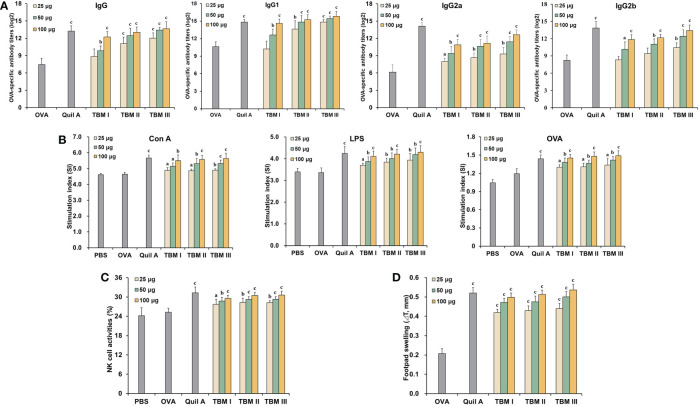
Tubeimosides potentiated humoral and cellular immune responses to OVA in mice. The serum and spleen were collected 2 weeks after the booster immunization. **(A)** Serum OVA-specific antibody titers by ELISA. **(B)** Splenocyte proliferation by the MTT method **(C)** NK cell activities by the MTT method. **(D)** DTH in the OVA-sensitized ICR mice. The data were presented as mean ± SD (*n* = 5). ^a^ *P* < 0.05, ^b^ *P* < 0.01, and ^c^ *P* < 0.001 *vs* OVA.

To evaluate the adjuvant effect of TBMs, OVA-specific antibody titers in the sera from the immunized ICR mice were first detected using ELISA. Serum OVA-specific IgG and IgG1 antibody titers in the mice immunized with OVA/Quil A and OVA/TBMs were markedly higher than those immunized with OVA alone (**Figure 2A**, *P* < 0.01 or *P* < 0.001). Noteworthy increases in serum OVA-specific IgG2a and IgG2b antibody levels were also observed in the mice co-immunized with TBMs and Quil A except for TBM I and TBM II (25 μg) towards IgG2b compared with OVA alone group (**Figure 2A**, *P* < 0.05, *P* < 0.01, or *P* < 0.001). The results suggested that all three TBMs boosted antigen-specific humoral response towards OVA in a dose-dependent manner.

Mitogen- and antigen-stimulated splenocyte proliferation as well as NK cell activity were detected using MTT method. Con A-, LPS-, and OVA-stimulated splenocyte proliferation in the mice immunized with OVA/Quil A and OVA/TBMs were markedly greater than that in the OVA alone group (**Figure 2B**, *P* < 0.05, *P* < 0.01, or *P* < 0.001). These results suggested that three TBMs promoted the activation of T and B cells in the immunized mice. The killing percentages of NK cells in splenocytes from the mice immunized with OVA/Quil A and OVA/TBMs toward K562 cells were dramatically higher than those immunized with OVA alone (**Figure 2C**, *P* < 0.05, *P* < 0.01, or *P* < 0.001), indicating that three TBMs intensified the cytotoxicity of NK cells in the immunized mice.

To further assess the CMI in mice induced by TBMs, DTH to OVA was also determined. The DTH to OVA in ICR mice sensitized with OVA/Quil A and OVA/TBM was markedly greater than that with OVA alone at 24 h after challenge (**Figure 2D**, *P* < 0.001). These results suggested that three TBMs augmented the CMI to OVA in mice.

Among three TBMs, the order in terms of inducing antibody responses, splenocyte stimulation index, and eliciting DTH was TBM III > TBM II > TBM I. However, no significant differences were observed in NK cell killing activities among three TBMs (*P* > 0.05).

### TBM I elicited both Th1/Th2 and Tc1/Tc2 responses towards OVA in mice

**Figure 3:**
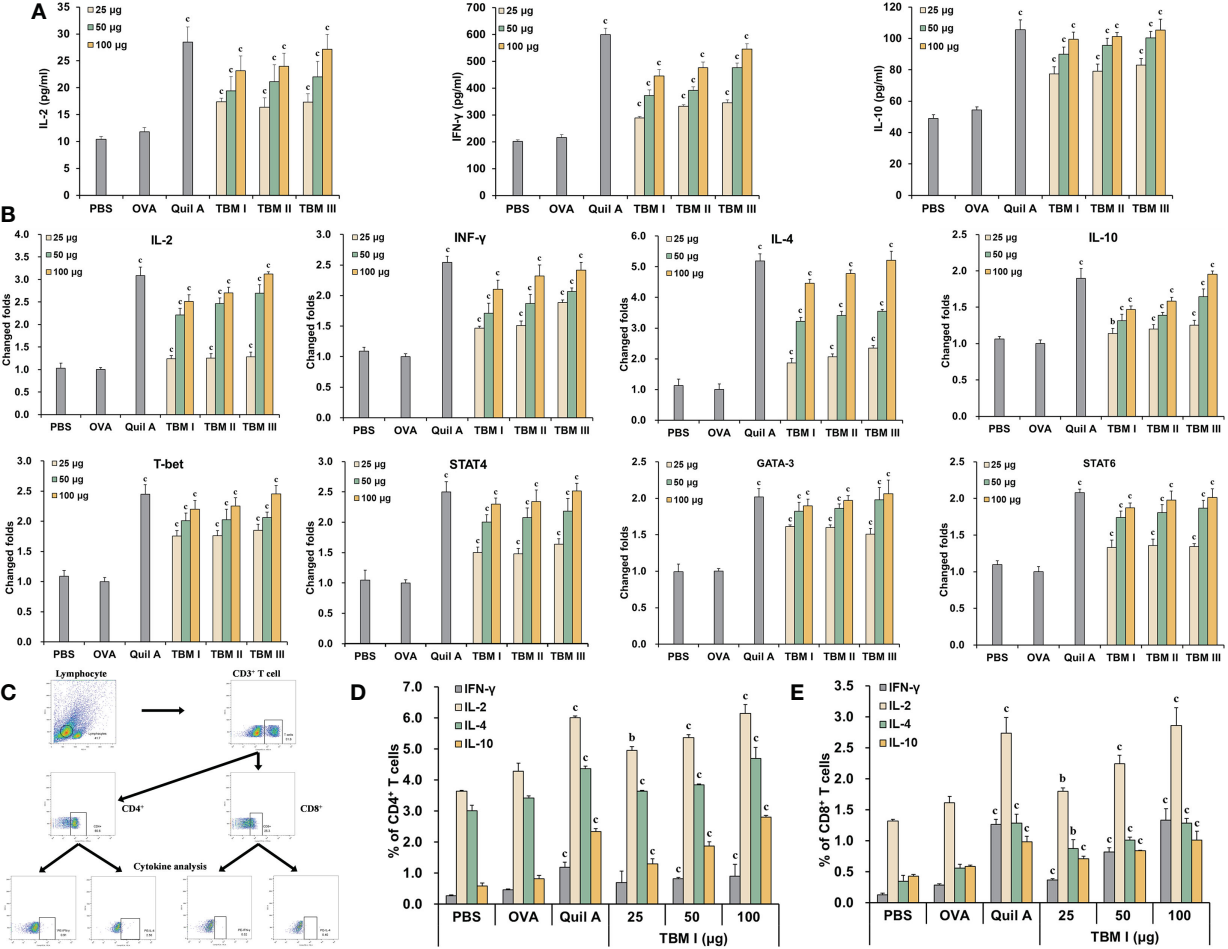
TBM I elicited both Th1/Th2 and Tc1/Tc2 responses towards OVA in mice. Splenocyte and culture supernatants were collected 72 h after stimulation with OVA. **(A)** The contents of cytokines in culture supernatants using ELISA kits. **(B)** The mRNA expression levels of cytokines and TFs in OVA-stimulated splenocytes by RT-qPCR. **(C–E)** Splenocytes were subjected to flow cytometry for measuring OVA-specific T cell cytokine response. **(C)** Schematic diagram of gating on IFN-γ^+^ and IL-4^+^ CD3^+^CD4^+^ and CD3^+^CD8^+^ T cells. Frequencies of OVA-specific CD4 **(D)** and CD8 **(E)** T cells expressing single cytokine. The data are presented as mean ± SD (*n* = 5). ^b^ *P* < 0.01, and ^c^ *P* < 0.001 *vs* OVA.

To explore the characteristics of the adjuvant action of TBMs, the contents of Th1 and Th2 cytokines in the culture supernatants of OVA-stimulated splenocytes were detected using ELISA kits. The levels of IL-2, IFN-γ, and IL-10 in the culture supernatants of OVA-stimulated splenocytes from the ICR mice immunized with OVA/Quil A and OVA/TBMs were prominently higher than those immunized with OVA alone (**Figure 3A**, *P* < 0.001). These results suggested that three TBMs dose-dependently triggered dual Th1 and Th2 responses to OVA in mice.

To elucidate the efficacy of TBMs on the Th1 and Th2 responses at the transcriptional level, the mRNA expression levels of cytokines and TFs in OVA-stimulated splenocytes from immunized ICR mice were determined by RT-qPCR. The mRNA levels of not only Th2 cytokines (IL-4 and IL-10) and TFs (GATA-3 and STAT6), but Th1 cytokines (IL-2 and IFN-γ) and TFs (T-bet and STAT4) in OVA-stimulated splenocytes from the immunized mice were dose-dependently markedly up-regulated by Quil A and TBMs compared with OVA alone group (**Figure 3B**, *P* < 0.01 or *P* < 0.001), suggesting that TBMs facilitated the gene expression of Th1 and Th2 cytokines and TFs in OVA-stimulated splenocytes from the immunized mice.

To further profile antigen-specific T cell cytokine response, the effects of TBM I on the intracellular cytokine responses in the splenocytes from the immunized C57BL/6J mice were also investigated using FCM through gating on CD3^+^CD4^+^ and CD3^+^CD8^+^ cells (**Figure 3C**). TBM I markedly increased OVA-specific Th1 (IFN-γ^+^ and IL-2^+^) and Th2 (IL-4^+^ and IL-10^+^) cells in OVA-stimulated splenocytes from the immunized mice at the suitable doses (**Figure 3D**, *P* < 0.01 or *P* < 0.001). Meanwhile, the frequencies of OVA-specific Tc1 (IFN-γ^+^ and IL-2^+^) and Tc2 (IL-4^+^ and IL-10^+^) cells in the OVA-stimulated splenocytes from the immunized mice were also markedly enhanced by TBM I compared to OVA alone group (**Figure 3E**, *P* < 0.01 or *P* < 0.001). These results suggested that TBM I elicited both Th1/Th2 and Tc1/Tc2 immune responses in OVA-immunized C57BL/6J mice.

### TBM I elicited the production of cytokines and chemokines at the injection site

**Figure 4:**
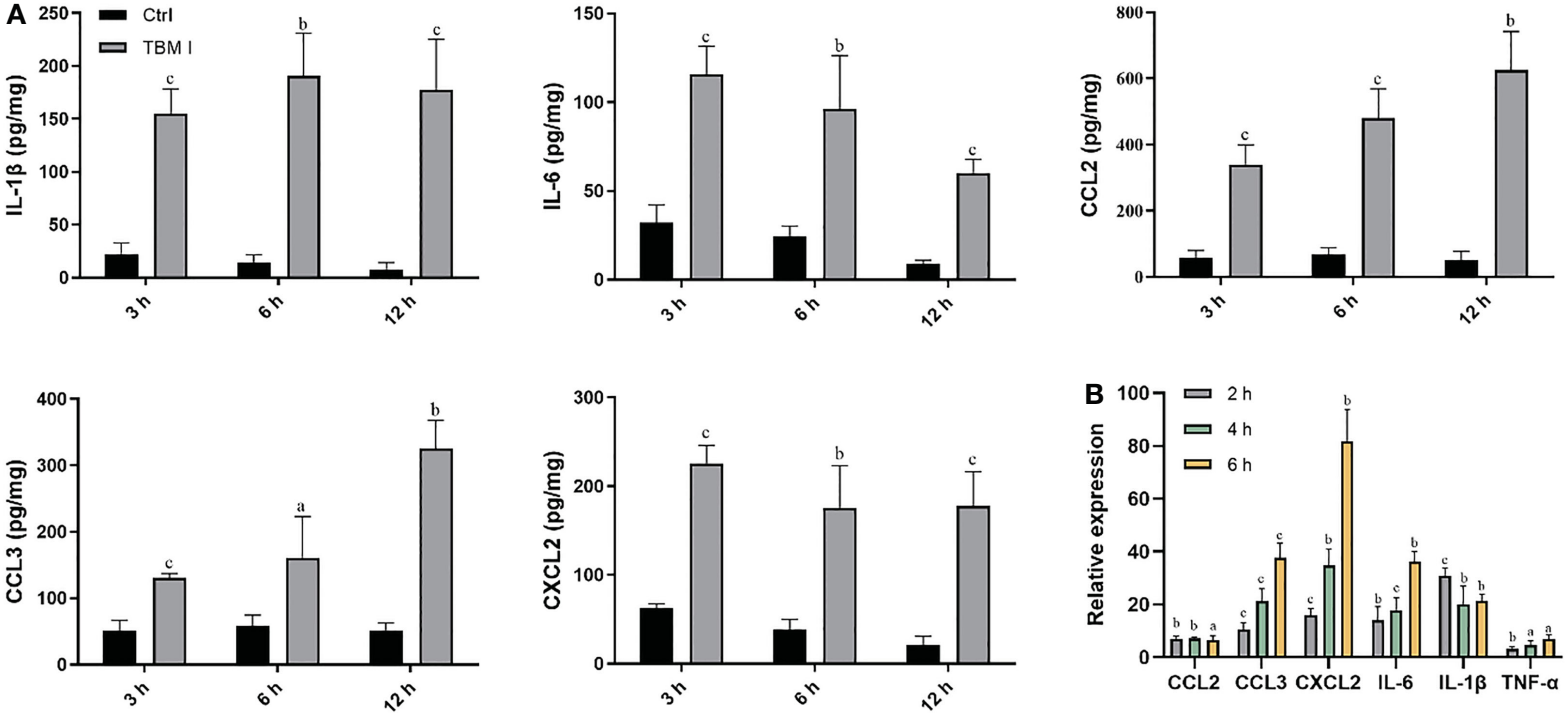
TBM I elicited cytokine and chemokine expression at the injection site. Mice were injected *i.m.* with TBM I and mock contralateral muscle received PBS. **(A)** The levels of cytokines and chemokines in quadriceps muscles at 3, 6, and 12 hpi by ELISA. **(B)** The gene expression levels of cytokines and chemokines in quadriceps muscles at 2, 4, and 6 hpi using RT-qPCR. The data are presented as mean ± SD (*n* = 5). ^a^ *P* < 0.05, ^b^ *P* < 0.01, and ^c^ *P* < 0.001 *vs* OVA.

The cytokines and chemokines play a key role in inducing the recruitment of various immune cells at the injection site. The levels of the cytokines (IL-1β and IL-6) and chemokines (CCL2, CCL3, and CXCL2) in the quadriceps muscles at the various times after injection were first examined by ELISA. TBM I led to a transient and broad release of different cytokines and chemokines in the injected quadriceps muscles. Cytokine levels began to increase in muscles within 3 hours post-injection (hpi), suggesting early production by resident cells. The levels of CCL2 and CCL3 continuously increased up to 12 hpi, while CXCL2 levels peaked at 3 hpi, and gradually declined at 6 hpi. Meanwhile, the elevated levels of classical pro-inflammatory cytokines IL-6 and IL-1β peaked at 6 hpi (**Figure 4A**), indicating the tendency of muscle tissues to recover.

The mRNA expression levels of cytokines and chemokines in the quadriceps muscles at the various times after injection were also examined by RT-qPCR. As shown in **Figure 4B**, TBM I induced early significant up-regulation of the mRNA expression of IL-1β, IL-6, and TNF-α, among which was most pronounced for IL-1β. The mRNA expression levels of CCL2, CCL3, and CXCL2 were also significantly up-regulated after TBM I injection (**Figure 4B**). These findings further confirmed that TBM I induced a rapid and transient inflammatory response at the injection site.

### TBM I promoted the recruitment and antigen uptake of immune cells at the injection site

**Figure 5:**
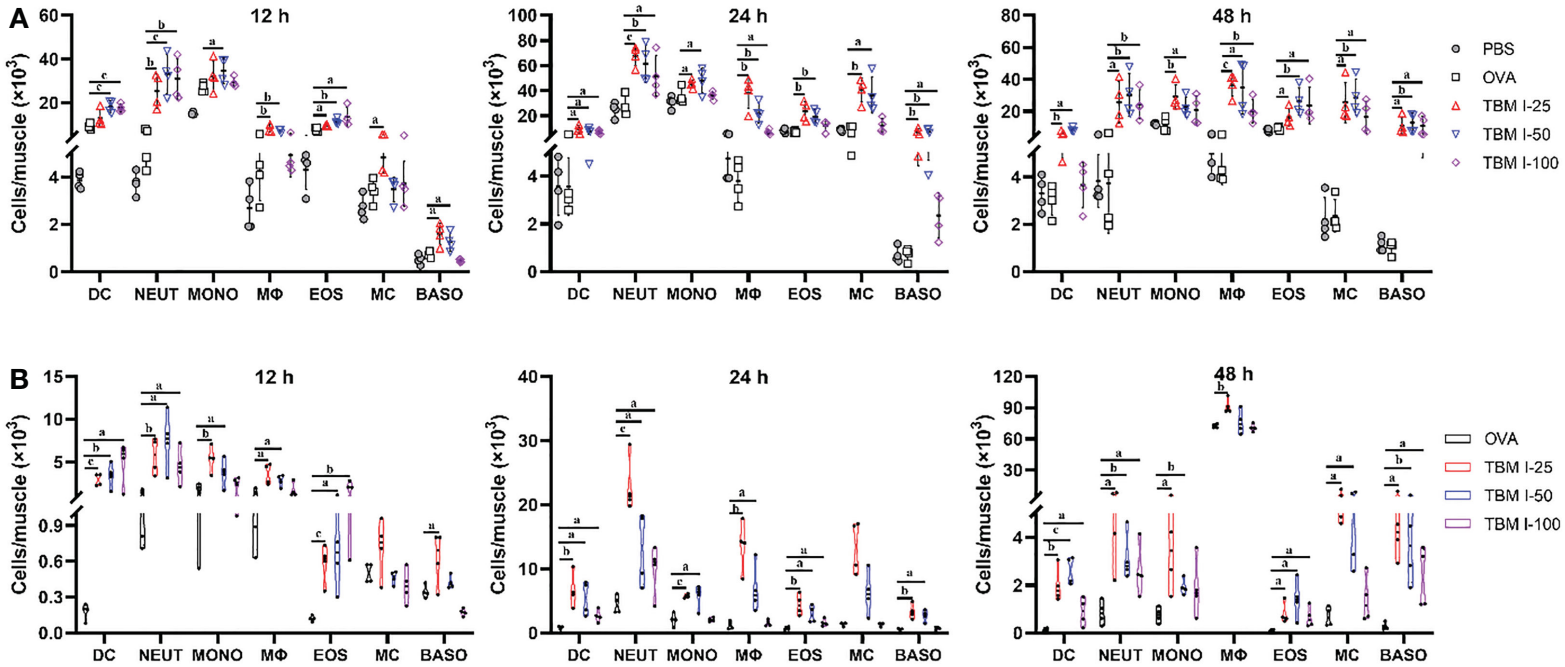
TBM I promoted the recruitment and antigen uptake of immune cells at the injection site. Mice were injected *i.m*. with OVA-AF488 alone or adjuvanted with TBM **(I)** The quadricep muscle tissues were collected at the indicated time points and subjected to FCM. **(A)** The number of immune cells recruited into the quadricep muscles at 12, 24, and 48 hpi. **(B)** The number of Ag^+^ immune cells recruited into the quadricep muscles at 12, 24, and 48 hpi. The data are presented as means ± SD (*n* = 4). ^a^ *P* < 0.05, ^b^ *P* < 0.01, and ^c^ *P* < 0.001 *vs* OVA-AF488. DC, dendritic cell; NEUT, neutrophil; MONO, monocyte; MΦ, macrophage; EOS, eosinophil; MC, mast cell; BASO, basophil.

The recruitment of innate immune cells to the injection site is a direct consequence of the local production of cytokines and chemokines. The muscle tissues were harvested at 12, 24, and 48 hpi, and the number of dendritic cells (DCs, CD11c^+^Ly-6C^−^Ly6G^−^), neutrophils (CD11c^−^Ly-6C^+^Ly6G^high^), monocytes (CD11c^−^Ly6G^−^Ly6C^+^), macrophages (F4/80^high^ CD3e^−^CD45R^−^), eosinophils (SigLecF^+^CD117^−^), basophils (SigLec F^−^FcER1^+^CD117^−^), and mast cell (SigLecF^−^CD117^+^FcER1^+^) were detected by FCM. Recruitment occurred specifically in the adjuvant co-injected muscles, while elevated cell numbers in the OVA-treated muscle were almost not found at any time point compared to the PBS-treated muscles, except for a relative higher extent of monocytes and eosinophils at 12 hpi (**Figure 5A**). At the early12 hpi, TBM I resulted in increased numbers of recruited cell types, particularly neutrophils and DCs, about 10-fold and 4-fold more cells as compared to the OVA-treated muscle, respectively (**Figure 5A**) . Neutrophils peaked at 24 hpi, with a subsequent decline at 48 hpi, whereas DCs and monocytes peaked at 12 hpi and decreased at 24 and 48 hpi (**Figures 5A**, **S1**). From 12 hpi onwards, however, TBM I resulted in a continuous increase of macrophages, eosinophils, basophils, and mastocytes, and a fairly increase in these cell numbers was observed at 48 hpi (**Figure 5A**). As for the dose-dependent analysis, 50 μg TBM I induced the optimized immune cell recruitment overall. Summarizing, TBM I induced a fast, but mostly transient, influx of neutrophils and DCs to the site of injection, followed by the recruitment of macrophages and monocytes, and finally eosinophils, basophils, and mastocytes (**Figure 5A**). The relatively high cell recruitment at the injection site induced by TBM I was observed at 24 hpi, indicating the peak of total cell infiltration.

The OVA-AF488 uptake of immune cells in the local tissues was also analyzed using FCM. As shown in **Figure 5B**, TBM I significantly promoted DCs, macrophages, monocytes, mast cells and neutrophils to uptake antigen. In contrast, only a few antigen-positive (Ag^+^) cell types were found in the muscles injected with OVA-AF488 alone. We extended the analysis to quantify the uptake of antigen to all identified cell types and to different time points after injection. Monocytes, macrophages, and dendritic cells but also neutrophils and mast cells were found to load antigen at all time points. Interestingly, we found that Ag^+^ DCs, Ag^+^ neutrophils, and Ag^+^ eosinophils peaked at 12 hpi for 100 μg TBM I, while for 25 μg and 50 μg TBM I these cells peaked at 24 hpi, indicating that the better adjuvant activity induced by a higher dose of TBM I presumably due to an earlier influx of Ag^+^ antigen-presenting cells (APCs), especially for DCs. At a later stage, TBM I resulted in higher Ag^+^ macrophage numbers compared to preceding time points, which was in accordance with the total cell kinetics (**Figure 5A**). These results indicated that TBM I promoted the recruitment and antigen uptake of immune cells at the injection site.

### TBM I induced the migration and antigen transport of immune cells to dLNs

**Figure 6:**
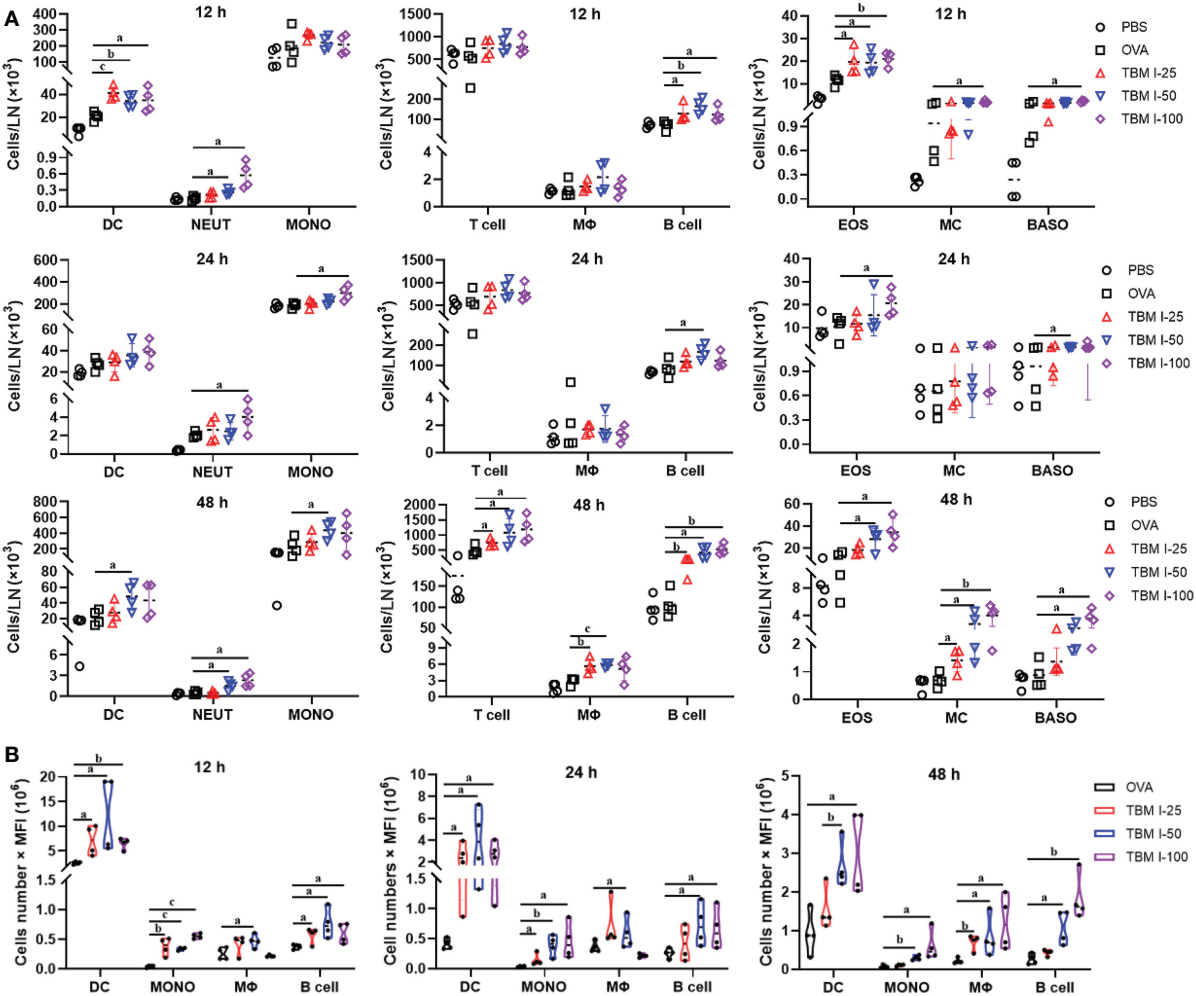
TBM I induced the migration of immune cells and antigen transport to dLNs. Mice were injected *i.m*. with OVA-AF488 alone or adjuvanted with TBM **(I)** The inguinal lymph nodes were collected at the indicated time point and subjected to FCM. **(A)** The number of immune cells migrated to dLNs at 12, 24, and 48 hpi. **(B)** The antigen ingestion of DCs, monocytes, macrophages, and B cells in dLNs at 12, 24, and 48 hpi by cell numbers × MFI. The data are presented as mean ± SD (*n* = 4). ^a^ *P* < 0.05, ^b^ *P* < 0.01, and ^c^ *P* < 0.001 *vs* OVA-AF488. DC, dendritic cell; NEUT, neutrophil; MONO, monocyte; MΦ, macrophage; EOS, eosinophil; MC, mast cell; BASO, basophil.

An acute inflammatory response and strong immune cell recruitment at the injection site led to enhanced antigen uptake and transport to dLNs, where Ag-specific T cell priming and production of Ag-specific, high affinity antibodies by B cells take place. To identify the cell types that serve as major antigen carriers induced by TBM I, the kinetics variation of the immune cell numbers in dLNs at the various times after injection was analyzed. As shown in **Figures 6A** and **S2**, TBM I significantly increased the numbers of DCs, neutrophils, B cells, and eosinophils at 12 hpi and 24 hpi, while at 48 hpi the numbers of macrophages, T cells, mast cells and basophils increased, indicating that complexed processes occurred continuously in the dLNs.

Furthermore, the relative contribution of different types of cells to antigen ingestion was also analyzed by multiplying cell numbers with median fluorescence intensity (MFI). The results showed that the OVA^+^ cells in the dLNs are mainly confined to four cell types namely DCs, macrophages, monocytes, and B cells while many various cells could carry antigen at the injection sites (**Figure 6B**). Overall, OVA alone led to slightly elevated numbers of Ag^+^ cells, whereas broad Ag-containing DCs were found in the dLNs, indicating that OVA alone could activate resident DCs only to some extent. In contrast, co-injection of OVA with TBM I led to dramatically elevated Ag-loading cells in the LNs, including DCs, monocytes, macrophages and B cells. Noticeably, Ag^+^ DCs peaked at 12 hpi or earlier and then gradually decreased over time, whereas Ag^+^ macrophages and B cells peaked at 48 hpi (**Figure 6B**). The Ag^+^ monocytes kept stable during 12-48 hpi. These findings indicated that TBM I facilitated the migration and antigen transport of immune cells to dLNs.

### TBM I regulated the expression of inflammatory response-related genes at injection site

**Figure 7:**
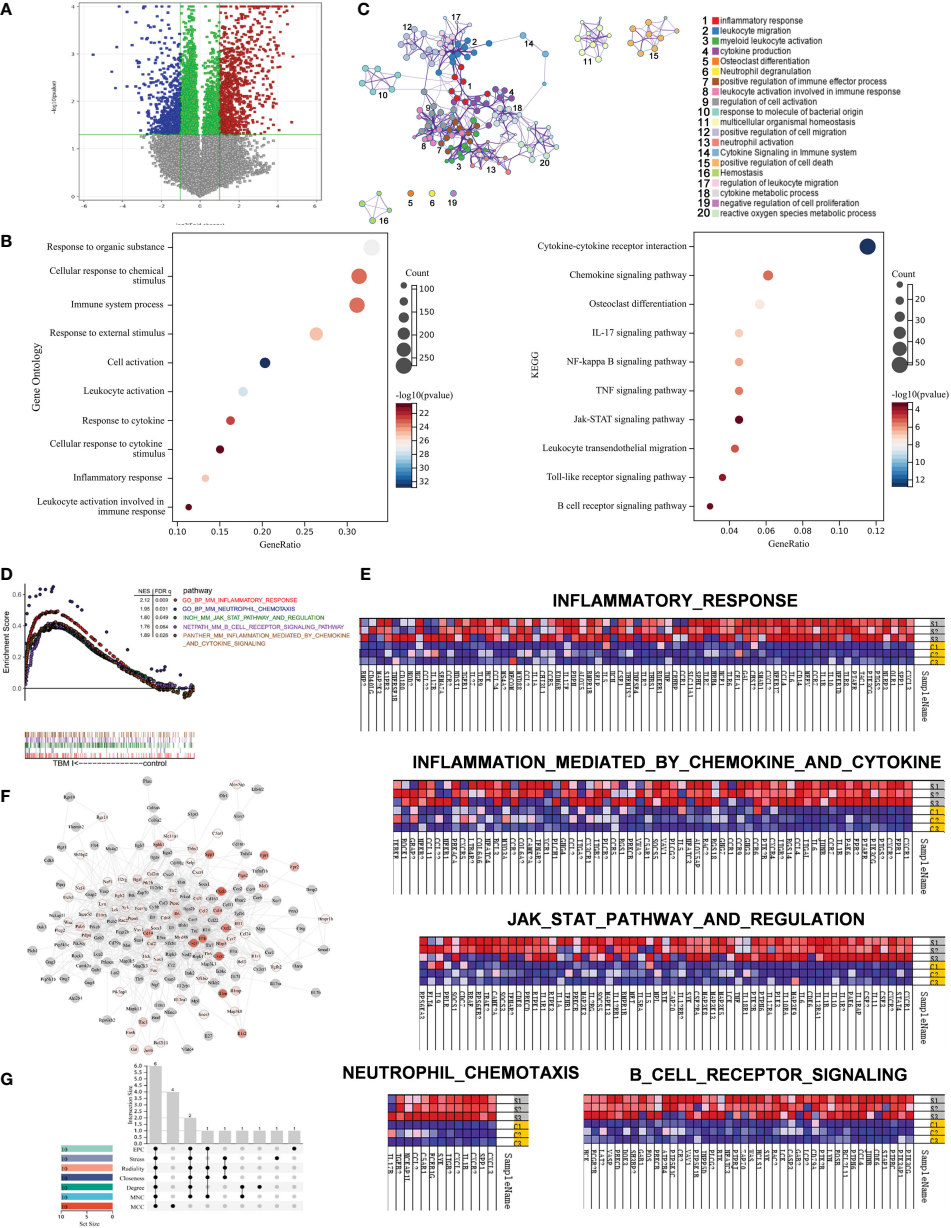
Gene set enrichment analysis of gene expression profiles in mouse quadricep muscles induced by TBM I and hub genes screening. **(A)** Volcano plots of gene expression in quadricep muscles. **(B)** GO function and KEGG pathway of DEGs. **(C)** Network of GO biological processes of DEGs. **(D)** Five leading-edge gene sets using GSEA. **(E)** Heatmap of enriched genes for each gene set. S1, S2, and S3 represent three replicates treated with TBM I C1, C2, and C3 represent three replicates of PBS control. **(F)** PPI network of core genes from GSEA. **(G)** The Upset plot of 6 overlapping hub genes by seven algorithms.

To further profile the transcriptional profiles, mouse quadricep muscles treated with TBM I for 4 h were subjected to SurePrint G3 mouse gene expression microarray. TBM I induced 1194 DEGs covering 654 up-regulated and 540 down-regulated genes (**Figure 7A**). These DEGs were subjected to the GO function and KEGG pathway enrichment analysis, and the results were shown in **Figure 7B**. The biological function was connected to cell activation (*P* = 1.35E-33), leukocyte activation (*P* = 1.51E-28), inflammatory response (*P* = 9.12E-26), immune system process (*P* = 5.72E-24), and response to cytokine (*P* = 3.36E-23). The most significant pathways were IL-17 signaling pathway (*P* = 1.36E-07), NF-κB signaling pathway (*P* = 6.64E-07), TNF signaling pathway (*P* = 3.09E-06), Toll-like receptor signaling pathway (*P* = 1.91E-04), and Jak-STAT signaling pathway (*P* = 6.62E-04), which are associated with inflammation. Meanwhile, a PPI network was generated to capture the relationships among the top 20 of enriched biological function using Metascape (**Figure 7C**). Most terms in the network were interrelated and involved in inflammation and immunity, further confirming that TBMS I could mediate the inflammatory and immune response in mouse quadricep muscles at injection site.

LESS related to “innate immune response” were further analyzed using GSEA. Five LESS such as “inflammatory response”, “inflammatory response mediated by cytokines and chemokines”, “JAK-STAT3 pathway and regulation”, “B cell receptor signaling”, and “neutrophil chemotaxis” were enriched in TBM I group (**Figure 7D**). The genes enriched in inflammatory response and neutrophil chemotaxis gene sets mainly included CXCL3, CXCL2, CCL2, CXCR2, CXCR4, CCR2, CCR5, IL6, PTGS2, and IL-17A, which contributed to the activation and mobilization of various innate immune cells including neutrophils, DCs, and macrophages, and the induction of Th17 immune response (**Figure 7E**, upper and lower left). In addition, TBM I also up-regulated the mRNA expression levels of TNF, IL-1β, IL5, IL6, CSF2, LCK and SYK, which are closely related to the JAK-STAT signaling pathway (**Figure 7E**, middle).

The PPI network of the immune-related genes from GSEA was constructed using Cytoscape. As shown in **Figure 7F**, 209 TBM I-specific core genes including 98 DEGs were involved in the network. Furthermore, the top 10 hub genes were calculated through the seven algorithms of plug-in cytoHubba, respectively (**Table S3**). Six common hub genes including IL-6, TNF, IL-1β, IL-10, SYK and LYN were obtained using upset plot (**Figure 7G**). Meanwhile, 10 TFs including NFKB1, RELA, STAT3, SPI1, CEBPA, SP1, JUN, STAT1, STAT6, and NR1I2 were predicted to mediate the expression of these core genes (**Table S4**).

### Key target analysis combined with network pharmacology and molecular docking analysis

**Figure 8:**
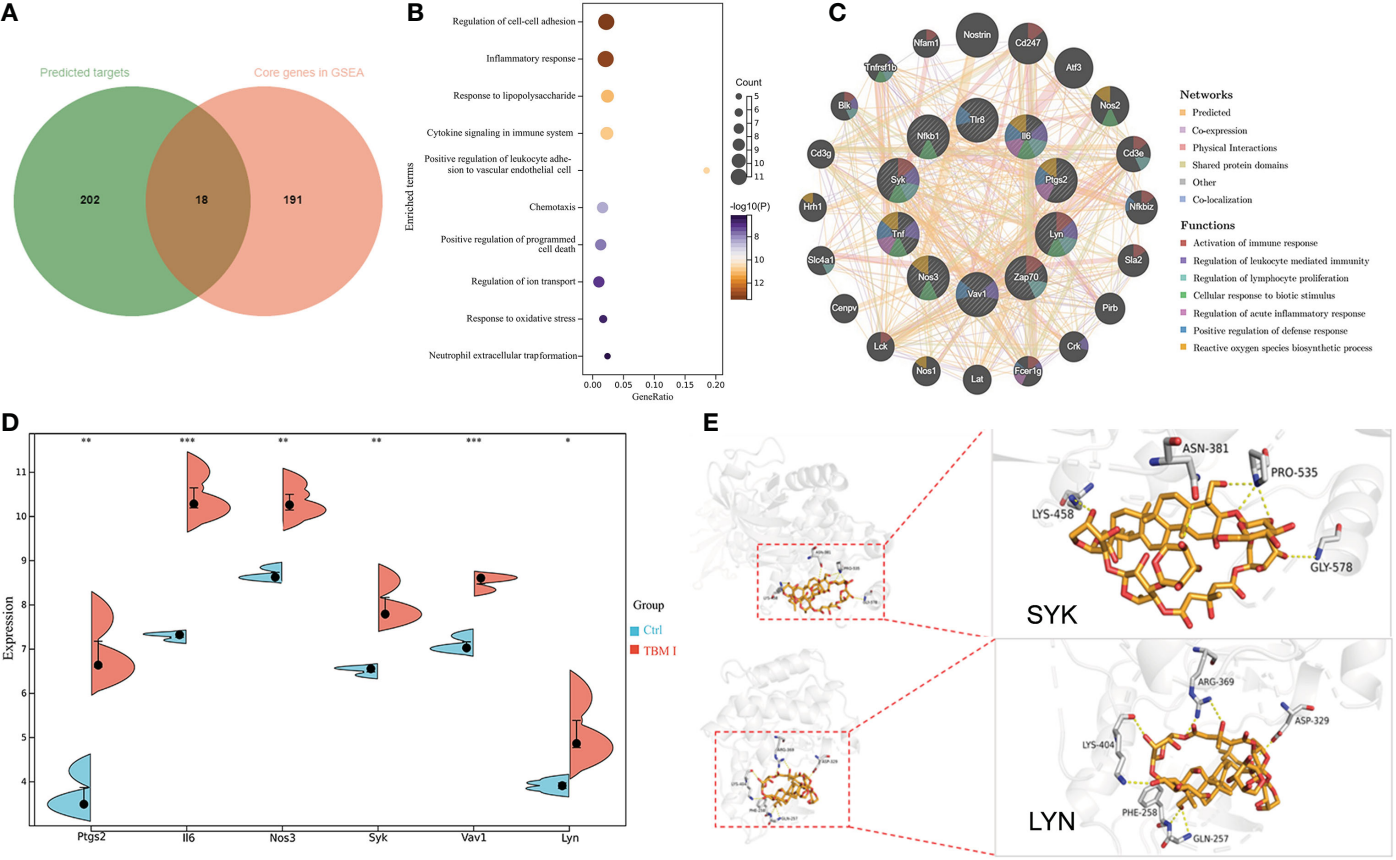
The predicted target of adjuvant activity of TBM I. **(A)** Overlap of therapeutic targets of TBM I (green) and core genes from GSEA of microarray data (orange). **(B)** Enriched terms of overlapped targets. **(C)** Key targets and their PPI network. **(D)** The expression level of key targets in microarray assay. The data are expressed as means ± SD (*n* = 3). ^*^ *P* < 0.05, ^**^ *P*< 0.01, and ^***^ *P* < 0.001 *vs* Ctrl. **(E)** Binding pattern diagram of TBM I and target protein SYK or LYN. The yellow dashed line represents the hydrogen bond interaction.

A total of 220 potential targets of TBM I were obtained by searching PubChem, Swiss Target Prediction, and SuperPred databases. Venn diagram showed that there were 18 common targets between 220 potential targets of TBM I and 209 core genes in the quadricep muscles induced by TBM I from GSEA (**Figure 8A**). The enrichment analysis of these common targets demonstrated that the pathways such as inflammatory response, cytokine signaling in immune system, cell adhesion, and cell chemotaxis were significantly enriched (**Figure 8B**), suggesting that TBM I induced inflammatory and immune response through regulating the expression of these targets. The PPI network of the common targets with scores greater than 0.4 was constructed using Cytoscape, which contained 18 nodes and 65 interaction pairs. PTGS2, IL-6, NOS3, SYK, VAV1, LYN, NFKB1, TLR8, ZAP70, and TNF were defined as 10 key targets using the MCODE plug-in of Cytoscape (**Table S5**). The co-expression network and related functions of these10 key targets were visualized using GeneMANIA database. Ten key targets constructed a complex PPI network with the predicted of 39.20%, co-expression of 22.92%, physical interactions of 17.59%, shared protein domains of 14.94%, other of 3.14%, and co-localization of 2.21% (**Figure 8C**). The function analysis showed that these targets were mainly involved in activation of immune response, regulation of leukocyte mediated immunity, regulation of lymphocyte proliferation, cellular response to biotic stimulus, regulation of acute inflammatory response, positive regulation of defense response, and reactive oxygen species biosynthetic process, suggesting that TBM I induced immune activation, inflammation, and defense response *via* these 10 targets. As shown in **Figure 8D**, microarray data revealed that TBM I significantly up-regulated the mRNA expression levels of 6 genes among10 key targets in quadricep muscles (*P* < 0.05, *P* < 0.01, or *P* < 0.001).

The molecular docking analysis was performed to further identify the direct targets of TBM I. Two candidate upstream targets SYK (PDB: 4FL3) and LYN (PDB: 2ZV7) which were both included in the hub genes from GSEA and located upstream of STAT were selected as the receptors for docking. The affinities between TBM I and proteins were -1.60 kcal/mol and -3.43 kcal/mol for SYK, and LYN, respectively. TBM I showed a closely binding conjugation with the protein active site through forming H bonds with the amino acid residues LYS-458, ASN-381, PRO-535 and GLY-578 of SYK, and LYS-404, ARG-369, ASP-329, GLN-257 and PHE-258 of LYN, respectively (**Figure 8E**), verifying the reliability of the screened targets based on network pharmacology integrated with transcriptome and suggesting the key role of SYK and LYN in mediating TBM I adjuvant activity.

### TBM I induced the inflammatory response in C2C12 cells through SYK–STAT3 signaling

**Figure 9:**
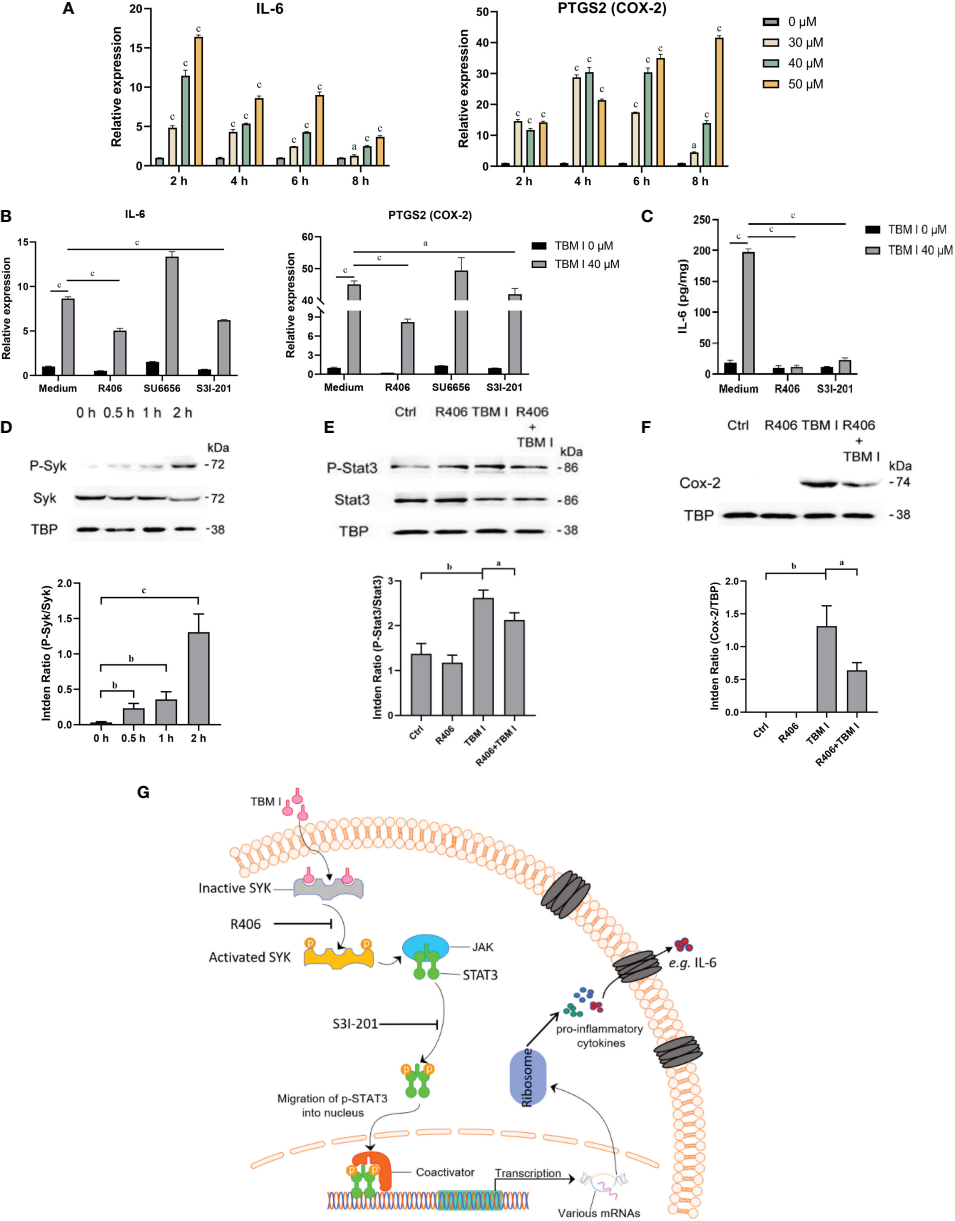
SYK-STAT3 pathway contributed the inflammatory response in C2C12 cells induced by TBM I. **(A)** The gene expression of IL-6 and PTGS2 in C2C12 cells induced by TBM I with RT-qPCR. **(B)** C2C12 cells were pretreated with SYK inhibitor (R406, 8 µM, 1 h), LYN inhibitor (SU6656, 2 μM, 1 h) or STAT3 inhibitor (S3I-201, 100 μM, 1 h) before TBM I (40 µM) stimulation for 4 h. The gene expression levels of IL-6 and PTGS2 in C2C12 cells were determined by RT-qPCR. **(C)** After pre-incubation with or without R406 (8 µM, 1 h) or S3I-201 (100 μM, 1 h), C2C12 cells were treated with medium or TBM I (40 μM) for 12 h. The level of IL-6 in the culture supernatants of C2C12 cells by ELISA. **(D)** C2C12 cells were treated with TBM I (40 μM) for 0, 0.5, 1, and 2 h, and the protein levels of P-Syk/SyK levels were detected by Western blotting. **(E)**, **(F)** After pre-incubation with or without R406 (8 µM, 1 h), C2C12 cells were treated with medium or TBM I (40 μM) for 4 h or 8 h, and the protein levels of P-Stat3/Stat3 **(E)** and COX-2 **(F)** were detected by Western blotting. The figure shown was representative of three independent experiments. Data were presented as mean ± SD (*n* = 3). ^a^ *P* < 0.05, ^b^ *P* < 0.01, and ^c^ *P* < 0.001. **(G)** Hypothetical pathway involving in the inflammatory response in C2C12 cells induced by TBM I.

C2C12 cells has been previously proved to be an *in vitro* ideal model for studying the mechanisms of action of SBAs for intramuscular vaccines. To validate the key role of two candidate targets SYK, LYN, and a key TF STAT3 in mediating the inflammatory response in C2C12 cells induced by TBM I, the effects of TBM I on the mRNA expression levels of IL-6 and PTGS2 (COX-2) were first analyzed using RT-qPCR. TBM I significantly up-regulated the mRNA expression levels of these two pro-inflammatory factors in the C2C12 cells in a time- and concentration-dependent manner (**Figure 9A**). R406 and S3I-201 were used to specifically inhibit the function of SYK and STAT3, respectively. The pretreatment with SYK inhibitor R406 and STAT3 inhibitor S3I-201 dramatically suppressed the up-regulated mRNA expression of IL-6 and PTGS2 in the C2C12 cells induced by TBM I, while pretreatment of LYN inhibitor SU6656 could not (**Figure 9B**). Moreover, the secretion of IL-6 from TBM I-treated C2C12 cells was also inhibited by R406 and S3I-201 (**Figure 9C**), suggesting that SYK and STAT3 involved in the pro-inflammatory responses in C2C12 cells induced by TBM I.

The effect of TBM I on the phosphorylation of SYK was detected using western blotting. TBM I significantly and time-dependently promoted the phosphorylation of SYK at as early as 0.5 h (**Figure 9D**). Furthermore, the pretreatment with R406 significantly suppressed the phosphorylation STAT3 (**Figure 9E**) and protein expression levels of COX-2 in TBM I-treated C2C12 cells (**Figure 9F**). These findings suggested that SYK regulated the activation of downstream STAT3 as well as gene and protein expression of pro-inflammatory factors in the C2C12 cells induced by TBM I.

## Discussion

Adjuvants affect the magnitude and type of adaptive immune response depending on the pathogens and immune responses required (3). An ideal adjuvant is requisite for the efficient humoral and cellular immune responses protecting against specific pathogens. According to the SAR of SBAs (15, 16), TBMs were supposed to have adjuvant potentials. In this study, three TBMs (**Figure 1**) were evaluated for the hemolysis, toxicity and adjuvant effects, as well as explored for their SAR and mechanisms of action.

Humoral immunity neutralizes and eliminates extracellular microorganisms and microbial toxins by producing specific antibodies. In this study, three TBMs boosted the serum OVA-specific IgG, IgG1, IgG2a, and IgG2b antibody responses in the immunized mice (**Figure 2A**). This result suggested that TBMs not only strengthened the immune response but also simultaneously triggered a Th1 and Th2 immune response to OVA.

Cellular immunity is effective in the process against infectious diseases, especially, in the clearance of intracellular pathogens and cancer cells (28). The proliferation assay showed that three TBMs remarkably augmented Con A-, LPS-, and OVA-stimulated splenocyte proliferation in the immunized mice (**Figure 2B**), revealing that three TBMs had the potential to elicit the activation of specific and nonspecific T- and B-lymphocytes in the immunized mice. NK cells release cytokines and lyse the infected cells to defend against tumor cells and intracellular pathogens (29). DTH reflects the antigen-specific T cell response and the immunologic memory. DTH has commonly been used to evaluate the CMI towards vaccines and the characteristic of adjuvants. In this study, three TBMs significantly promoted the cytolytic activities of NK cells in the immunized mice (**Figure 2C**) and the DTH to OVA in mice (**Figure 2D**). These results revealed that TBMs were highly efficient in eliciting the cellular immune responses.

Once activated, CD4 T cells differentiate into different subsets and then form a cytokine environment that regulates the outcome of adaptive immunity. Th1 and Th2 cells control and eliminate intracellular and extracellular pathogens, respectively. Th1 cells are characteristic of producing IFN-γ and IL-2 accompanied by the production of mouse IgG2a, IgG2b and IgG3 antibodies involved in phagocytosis and opsonization events. Th2 cells are typical of secreting IL-4 and IL-10 cytokines, which stimulate the production of IgM and IgG1 in mice (30, 31). Adjuvants facilitate the production of Th1 and/or Th2 cytokines resulting in Th1 and/or Th2 immune responses. Three TBM significantly induced the secretion of not only Th2 cytokine (IL-10) but also Th1 cytokines (IFN-γ and IL-2) from OVA-stimulated splenocytes in the immunized mice (**Figure 3A**). All three TBMs also remarkably facilitated the mRNA expression of not only Th2 cytokines (IL-4 and IL-10) and TFs (GATA-3 and STAT6), but also Th1 cytokines (IFN-γ and IL-2) and TFs (T-bet and STAT4) in OVA-stimulated splenocytes (**Figure 3B**). These findings suggested that three TBMs simultaneously elicited Th1 and Th2 type immune responses to OVA in mice.

The epidemics infected by the complicated pathogens require CMI leading to the activation and differentiation of effector CD4 and CD8 T cells. After activation by the cytokines secreted from CD4 T cells, macrophages eliminate phagocytized pathogens and limit the survival and spreading of pathogens. Cytokines from CD4 T cells also prime the antigen-specific B cells to polarize into antibody-secreting and memory B cells. CD8 cells secrete cytotoxic factors to target pathogens in infected or transformed cells (32). To further clarify the antigen-specific T cell responses of the splenocytes in the immunized mice induced by TBM I, the intracellular cytokines in CD4 and CD8 T cells were analyzed by FCM (**Figure 3C**). TBM I miraculously raised antigen-specific IL-2^+^, IFN-γ^+^, IL-4^+^, and IL-10^+^ CD4 T cells in OVA-stimulated splenocytes of the immunized mice (**Figure 3D**), which was coincident with its strong induction of both Th1 and Th2 immune responses. Furthermore, TBM I also lifted OVA-specific Tc1 (IL-2^+^ and IFN-γ^+^) and Tc2 (IL-4^+^ and IL-10^+^) CD8 T cells in splenocytes from the OVA-immunized mice (**Figure 3D**), indicating its potentials for eliciting simultaneous Tc1/Tc2 immune response.

Although adjuvants have been widely employed in vaccine compositions, little is well understood about their precise mechanisms. SBAs might shape the adaptive immune responses by improving a local innate immune microenvironment. The distribution and duration of early inflammatory events are crucial in the development of antigen-specific adaptive immunity in the forms of antibody and/or T cells (33, 34). SBAs induced the production of cytokines and chemokines in local tissues. The immune cells mobilization, in turn, is most likely induced by distinct cytokines and chemokines in a local adjuvant-specific micro-milieu (17). TBM I induced a pro-inflammatory profile with a rapid and transient expression of IL-1β, IL-6, CCL2, CCL3, and CXCL2 at the injection site as early as 3 hpi (**Figure 4**). This cell recruitment mechanism promoted by adjuvant is the basis for the generation of inflammation at the inoculation site, providing a high cellular influx and prolonging the antigen bond, consequently enhancing the interaction between antigen and APCs (35).

Sustainable cellular recruitment is essential to trigger an effective and on-going immune response (36). To elucidate the formation of an immune microenvironment, the quantification of cell infiltrate was performed after injection into the quadriceps muscles with antigen alone or together with TBM I. The results showed that TBM I stood out showing more cell mobilization at 12, 24 and 48 hpi, pointing out that TBM I had a higher capacity to promote cellular recruitment, triggering the innate immune response at the injection site (**Figure 5A**). It could be observed that there was a notable decrease in monocyte numbers at 48 hpi, while the number of macrophages significantly increased, which may be caused by the monocyte–macrophage conversion.

The superior immune responses might be ascribed to sustainable antigen release and robust antigen uptake and transport, which promoted a series of cascade reactions, including enhanced DCs maturation, increased lymphocytes activation and augmented follicular helper CD4 T cells differentiation in dLNs (37). SBAs have been proven to enhance antigen uptake and induce DCs activation in dLNs, resulting in a subsequent strong antibody and T-cell response (38). The effect of TBM I on the antigen ingestion of all identified cell types in the injection site and dLNs was analyzed. The capacity of TBM I to enhance antigen capture was apparent at all time points, whereas OVA alone showed relatively few Ag^+^ cells, and a decrease was observed from 24 hpi onwards (**Figures 5B**, **6B**). This corroborates other studies showing that adjuvants prolong the retention of antigen in the injection site and/or the dLNs (39, 40). The results showed high numbers of Ag^+^ macrophages in the injection site at 48 hpi (**Figure 6B**), which reconfirmed the monocyte–macrophage differentiation. Interestingly, the individual antigen uptake of the relatively few recruited DCs was most potent in draining LNs (**Figure 6B**). This may be explained in this way as another study has demonstrated that saponins had an early effect on macrophage migration to LNs and macrophages played an intermediate role in promoting DCs maturation (41). Overall, the antigen ingestion and translocation from the inoculum site to the dLNs are relevant and contribute to the development of an adaptive immune response.

To further explore the mechanism of adjuvant activity of TBM I, the transcriptome of the quadriceps muscles injected with TBM I was analyzed using microarray and whole genes were subjected to GSEA to screen the core genes induced by TBM I. The integrating analysis of the network pharmacology computational prediction and TBM I-specific core genes was conducted to reveal 18 potential targets of TBM I (**Figure 8A**). These 18 overlapping targets were involved in inflammatory response, cell adhesion, and chemotaxis (**Figure 8B**), suggesting that TMB I induced sterile inflammation in the injected muscle tissues through regulating these targets. Sterile inflammation leads to the canonical transendothelial infiltration of leukocytes from the blood. Leukocyte traffic in focal sterile injury follows by the sequence of recruitment of macrophages, neutrophils, and inflammatory monocytes (42), which is of importance in initiating innate immune response. PTGS2, IL-6, NOS3, SYK, VAV1, LYN, NFKB1, TLR8, ZAP70, and TNF were further defined as 10 key candidate targets, which were related to the immune activation, acute inflammatory, and defense response induced by TBM I (**Figure 8C**). VAV1 and ZAP70 are both positive regulators of T cell activation, regulate motility, adhesion and cytokine expression of mature T-cells (43). PTGS2 is involved in the inflammatory response through activating MAPK pathway to promote inflammatory response (44). IL-6 and TNF are typical proinflammatory cytokines leading to the development and progression of inflammation. Besides, the expression and activation of key TF STAT3 can be triggered by the activated immunological circuit involving innate lymphoid cells (45).

Furthermore, the molecular docking results showed that TBM I successfully docked with two upstream proteins SYK and LYN (**Figure 8E**). SYK, a nonreceptor protein tyrosine kinase, has recently been shown to relay adaptive and innate immune signaling (46) and regulate STAT3 activity (47). LYN, the Src-family kinase, is an important regulator of immunoreceptor signal transduction, initiating pro-inflammatory and suppressive signaling pathways in myeloid immune cells and in B lymphocytes (48). These findings suggested that TBM I could interact with SYK, and/or LYN to induce innate immunity, consequently influencing adaptive immune responses. Further inhibitor assay and validation experiments revealed that TBM I induced the phosphorylation of SYK in C2C12 cells. The phosphorylated SYK further induces phosphorylation of downstream STAT3, and the phosphorylated STAT3 is then transferred into the nucleus to bind to the target sequence on DNA. Finally, the transcription of immune response genes such as IL-6 and PTGS2 is enhanced, leading to an inflammatory response (**Figure 9G**).

TBM I, II, and III shared a similar skeleton and two sugar chains at positions C-3 and C-28 with a dicrotalic acid bridge. The structure of TBM II differs from that of TBM I by the addition of one hydroxyl at C-16. TBM III and TBM II only differ from each other by the terminal glycosyl at the C-3 sugar chain with *α*-L-arabinopyranosyl and *β*-D-glucopyranosyl, respectively. Among three TBMs, the order of the adjuvant activity was TBM III > TBM II > TBM I. These findings suggested that the hydroxyl at C-16 strengthened the adjuvant potency and that the terminal hexose in the C-3 sugar chain might be crucial to the adjuvant activities of TBMs.

The order of hemolytic activities of three TBMs in terms of the HD_50_ values was TBM III > TBM I > TBM II. However, TBM II is more lethal than TBM I and TBM III to mice. Although there were no significant differences in hemolysis among Quil A, TBM I and TBM III, the lethality of Quil A to mice was far greater than that of the two latter. These results indicated that there is no necessary relationship between hemolytic activity and toxicity of saponins, which might depend on the type of saponins. Therefore, TBMs could be modified structurally to develop various structural analogs with a stronger adjuvant effect and lower hemolytic activity and toxicity.

In conclusion, it was for the first time demonstrated that TBMs possessed the adjuvant effects to boost the magnitude of immune responses and improve the quality of the immune responses. In mechanisms, TBM I could exert the adjuvant activity *via* SYK-STAT3 axis to induce proinflammatory response in the muscle cells. The SAR information of TBMs contributed to semisynthetic development of safe and efficient saponin adjuvants.

## Data availability statement

The data presented in the study are deposited in the GEO repository, accession number GSE205650.

## Ethics statement

The animal study was reviewed and approved by Experimental Animal Ethics Committee of Zhejiang University.

## Author contributions

ZH: Experimental performance, data acquisition and analysis, bioinformatic analysis, and drafting the manuscript. JJ, XC, and YH: Experimental performance, data acquisition. HS: Design of the study, experimental performance, data analysis, and finalizing manuscript. All authors contributed to manuscript revision, read, and approved the submitted version. All authors contributed to the article and approved the submitted version.
