# Supplementary material for: Adjuvant activity of tubeimosides by mediating the local immune microenvironment
Source: Front Immunol. 2023 Feb 10;14:1108244. doi: 10.3389/fimmu.2023.1108244 (PMC9950507; doi:10.3389/fimmu.2023.1108244)
Supplement: Supplementary file 1 [file DataSheet_1.docx]

Supplementary Material

Article Title

Ziyi Han, Junjie Jin, Xiangfeng Chen, Yanfei He, Hongxiang Sun*

**^*^ Correspondence:** Hongxiang Sun: [sunhx@zju.edu.cn](mailto:sunhx@zju.edu.cn)

**Supplementary Figure 1.** FACS dot plot of immune cells in the injected muscles at 24 hpi.

**Supplementary Figure 2.** FACS dot plot of immune cells in dLNs at 24 hpi.

**Supplementary Table 1.** ^1^C- and ^13^C-NMR data of tubeimosides.

**Supplementary Table 2.** Primers used for RT-qPCR.

**Supplementary Table 3.** The top 10 hub genes rank in cytoHubba.

**Supplementary Table 4.** Predicted key transcriptional factors (TFs).

**Supplementary Table 5.** The details of the key targets.


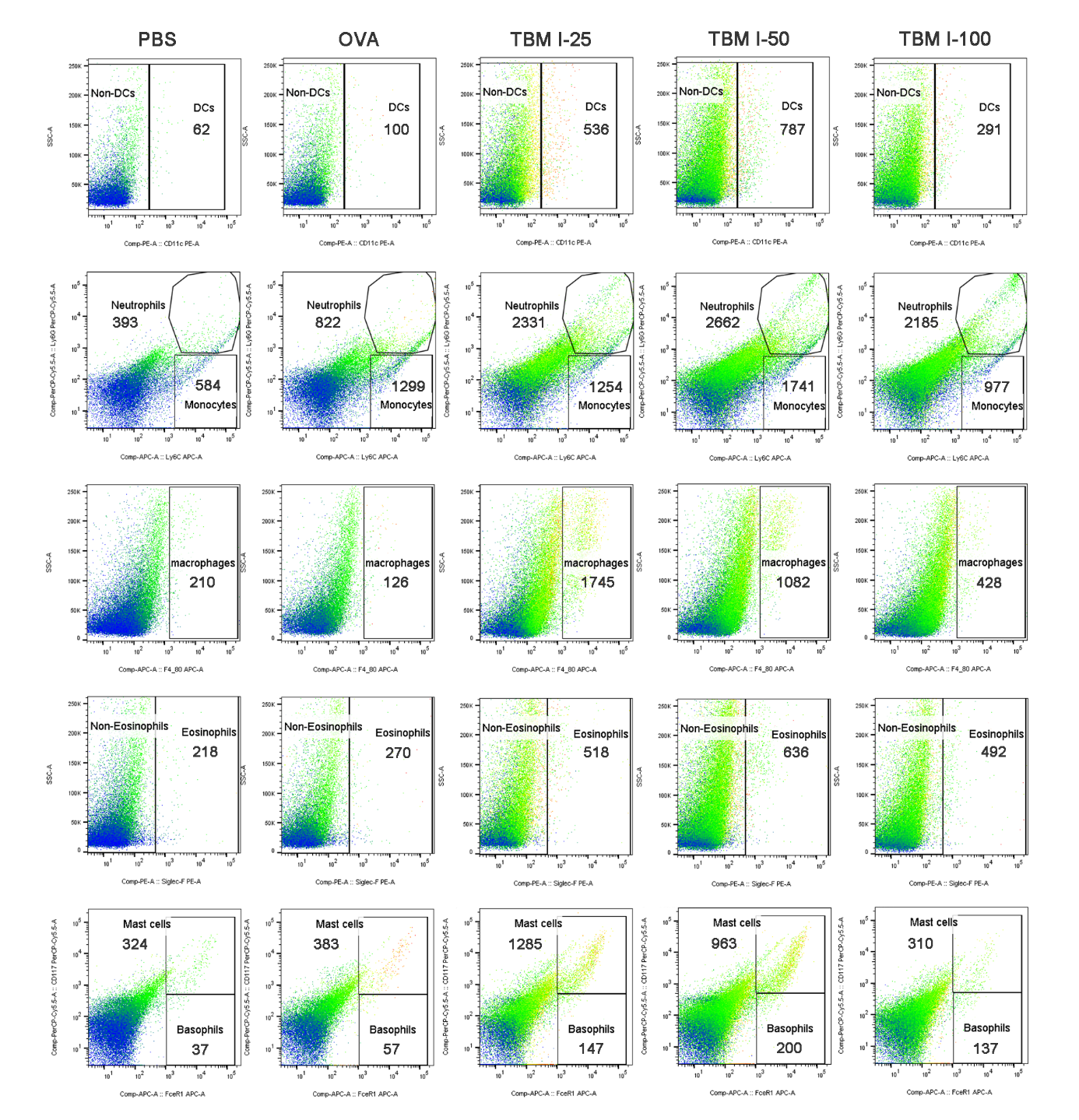
**Supplementary Figure 1.** FACS dot plot of immune cells in the injected muscles at 24 hpi. The figures tagged represent the cell numbers for immediate analysis.


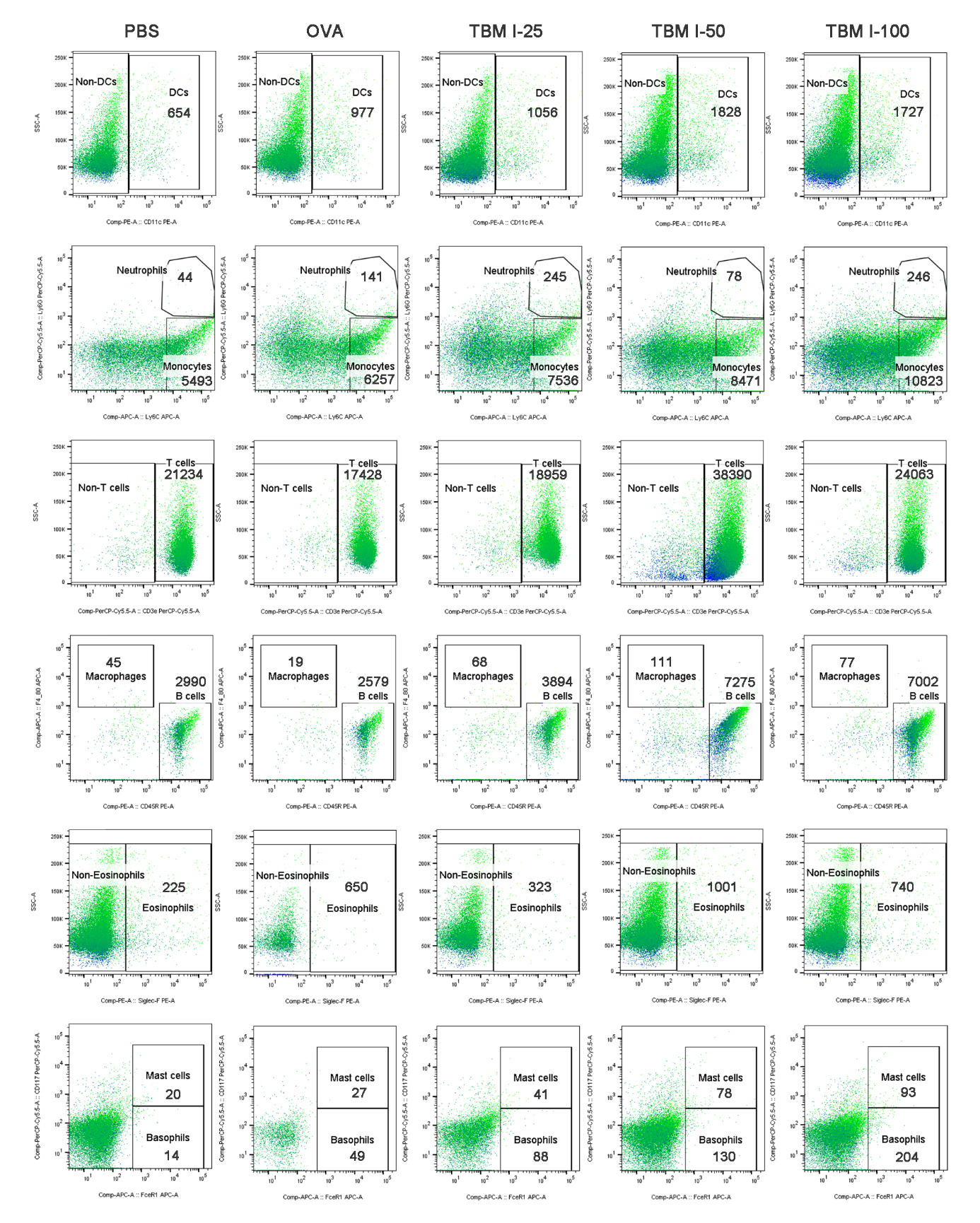
**Supplementary Figure 2.** FACS dot plot of immune cells in dLNs at 24 hpi. The figures tagged represent the cell numbers for immediate analysis.

**Supplementary Table 1.** ^13^C-NMR Data of tubeimosides.

|  | TBM I | | TBM II | | TBM III | |
| --- | --- | --- | --- | --- | --- | --- |
|  | *δ*_C_ | *δ*_H_ | *δ*c | *δ*H | *δ*c | *δ*H |
| Aglycone |  |  |  |  |  |  |
| 1 | 44.01 | 2.14, 1.32 | 43.71 | 2.05, 1.27 | 44.09 | 2.34, 1.28 |
| 2 | 69.22 | 4.62 | 69.51 | 4.45 | 69.85 | 4.79 |
| 3 | 84.99 | 4.30 | 83.77 | 4.24 | 82.55 | 4.26 |
| 4 | 43.57 | – | 42.75 | – | 42.61 | – |
| 5 | 48.17 | 1.84 | 46.61 | 1.81 | 47.61 | 1.92 |
| 6 | 19.96 | 2. 06, 1.78 | 18.78 | 2.03, 1.85 | 18.78 | 2.23, 1.95 |
| 7 | 34.87 | 1.67, 1.64 | 33.22 | nd, 1.54 | 33.20 | 1.99, 1.96 |
| 8 | 41.22 | – | 39.45 | – | 40.19 | – |
| 9 | 48.55 | 1.81 | 47.81 | 1.88 | 46.67 | 2.05 |
| 10 | 39.34 | – | 37.93 | – | 35.89 | – |
| 11 | 20.50 | 2.09, 1.98 | 24.18 | 2.08, 1.98 | 24.10 | 2.25, 2.18 |
| 12 | 125.27 | 5.37 | 121.72 | 5.42 | 124.63 | 5.79 |
| 13 | 146.50 | – | 143.65 | – | 146.64 | – |
| 14 | 42.05 | – | 42.20 | – | 42.13 | – |
| 15 | 28.18 | 1.98,1.21 | 35.97 | 2.11, 1.64 | 38.03 | 2.21, 1.93 |
| 16 | 20.18 | 2.10, 1.98 | 75.07 | 5.21 | 73.40 | 5.26 |
| 17 | 49.01 | – | 49.38 | – | 49.24 | – |
| 18 | 42.01 | 3.19 | 41.51 | 3.36 | 39.02 | 3.51 |
| 19 | 46.48 | 1.72, 1.24 | 46.57 | 2.70, 1.20 | 46.66 | 2.81, 1.40 |
| 20 | 30.76 | – | 30.58 | – | 30.25 | – |
| 21 | 34.35 | 1.38,1.14 | 35.96 | 2.32, 1.12 | 35.73 | 2.41, 1.28 |
| 22 | 32.61 | 1.80, 1.77 | 32.87 | 2.24, 1.95 | 33.30 | 2.38, 2.07 |
| 23 | 64.24 | 4.32, 3.69(nd) | 64.44 | 4.08, 3.54 | 64.03 | 4.36, 3.76 |
| 24 | 15.45 | 1.52 | 15.59 | 1.45 | 15.53 | 1.46 |
| 25 | 17.72 | 1.58 | 17.47 | 1.53 | 17.34 | 1.69 |
| 26 | 17.75 | 1.14 | 17.85 | 1.04 | 17.48 | 1.36 |
| 27 | 26.05 | 1.21 | 27.23 | 1.63 | 27.09 | 1.94 |
| 28 | 178.01 | – | 175.84 | – | 177.56 | – |
| 29 | 33.43 | 0.90 | 33.19 | 0.86 | 34.19 | 1.02 |
| 30 | 20.45 | 0.90 | 23.19 | 0.91 | 24.62 | 1.05 |

**Supplementary Table 1.** ^13^C-NMR Data of tubeimosides (continued).

|  | TBM I | | | TBM II | | | TBM III | |
| --- | --- | --- | --- | --- | --- | --- | --- | --- |
|  | *δ_C_* | *δ_H_* | | *δ_C_* | *δ_H_* | | *δ_C_* | *δ_H_* |
| Acyl moiety |  |  |  | |  |  | |  |
| 1 | 173.05 | – | 171.27 | | – | 173.05 | | – |
| 2 | 50.23 | 3.45, 3.03 | 47.20 | | 3.31, 2.86 | 48.52 | | 3.62, 2.95 |
| 3 | 69.89 | – | 69.97 | | – | 70.27 | | – |
| 4 | 49.91 | 3.30, 3.04 | 47.36 | | 3.16, 2.93 | 49.23 | | 3.22, 2.70 |
| 5 | 173.16 | – | 172.96 | | – | 173.41 | | – |
| 3-Me | 26.58 | 1.98 | 26.53 | | 1.87 | 26.08 | | 1.81 |
| 3-O-Glc |  |  | |  |  | |  |  |
| 1 | 105.23 | 5.02 | | 103.82 | 4.91 | | 105.73 | 5.07 |
| 2 | 81.91 | 4.25 | | 79.76 | 4.14 | | 83.73 | 4.08 |
| 3 | 80.44 | 4.18 | | 79.09 | 3.98 | | 78.20 | 4.23 |
| 4 | 73.88 | 4.10 | | 71.78 | 3.95 | | 70.27 | 4.13 |
| 5 | 79.20 | 3.80 | | 78.64 | 3.64 | | 77.3 | 3.81 |
| 6 | 64.12 | 4.42, 4.25 | | 62.56 | 4.27, 4.12 | | 62.34 | 4.41, 4.23 |
| Ara |  |  | |  |  | |  |  |
| 1 | 105.50 | 5.54 | | 105.57 | 5.42 | |  |  |
| 2 | 76.21 | 4.39 | | 73.91 | 4.29 | |  |  |
| 3 | 73.27 | 4.22 | | 73.25 | 4.10 | |  |  |
| 4 | 73.31 | 5.52 | | 72.55 | 5.40 | |  |  |
| 5 | 64.66 | 4.06, 3.57 | | 64.44 | 3.95, 3.46 | |  |  |
| Glc |  |  | |  |  | |  |  |
|  |  |  | |  |  | | 106.61 | 5.24 |
|  |  |  | |  |  | | 77.43 | 4.08 |
|  |  |  | |  |  | | 77.81 | 4.13 |
|  |  |  | |  |  | | 70.30 | 4.23 |
|  |  |  | |  |  | | 75.66 | 4.02 |
|  |  |  | |  |  | | 65.85 | 4.98, 4.62 |

**Supplementary Table 1.** ^13^C-NMR Data of tubeimosides (continued).

|  | TBM I | | TBM II | | TBM III | |
| --- | --- | --- | --- | --- | --- | --- |
|  | *δ_C_* | *δ_H_* | *δ_C_* | *δ_H_* | *δ_C_* | *δ_H_* |
| 28-O-Ara |  |  |  |  |  |  |
| 1 | 95.21 | 6.14 | 93.89 | 6.05 | 95.91 | 5.81 |
| 2 | 75.16 | 4.63 | 77.59 | 4.43 | 76.31 | 4.63 |
| 3 | 71.75 | 4.44 | 75.34 | 4.33 | 74.98 | 4.13 |
| 4 | 67.55 | 4.25 | 69.51 | 4.10 | 69.83 | 4.07 |
| 5 | 66.29 | 4.32, 3.80 | 68.65 | 4.24, 3.67 | 67.36 | 4.16, 3.75 |
| Rha |  |  |  |  |  |  |
| 1 | 100.75 | 6.13 | 100.9 | 5.94 | 103.09 | 6.31 |
| 2 | 73.29 | 4.92 | 72.55 | 4.81 | 72.94 | 5.03 |
| 3 | 79.68 | 4.57 | 77.81 | 4.43 | 78.19 | 4.65 |
| 4 | 76.16 | 6.05 | 75.61 | 5.91 | 73.42 | 6.05 |
| 5 | 68.65 | 4.33 | 67.34 | 4.23 | 68.06 | 4.48 |
| 6 | 19.78 | 1.50 | 17.86 | 1.32 | 18.13 | 1.45 |
| Xyl |  |  |  |  |  |  |
| 1 | 108.04 | 5.01 | 106.62 | 4.86 | 107.43 | 5.10 |
| 2 | 75.11 | 3.80 | 75.62 | 3.64 | 74.60 | 3.79 |
| 3 | 79.77 | 4.06 | 77.60 | 3.95 | 77.81 | 4.08 |
| 4 | 72.55 | 4.08 | 71.78 | 3.93 | 70.29 | 4.13 |
| 5 | 67.58 | 4.10, 3.60 | 67.15 | 3.94, 3.42 | 67.39 | 4.16, 3.65 |

**Supplementary Table 2.** Primers used for RT-qPCR.

| Gene | Primer sequence | Product size (bp) |
| --- | --- | --- |
| GAPDH | 5’-AGCCTCGTCCCGTAGACAA-3’  5’-AATCTCCACTTTGCCACTGC-3’ | 104 |
| IL-2 | 5’-CCCAAGCAGGCCACAGAATTGAAA-3’  5’-AGTCAAATCCAGAACATGCCGCAG-3’ | 81 |
| IFN-γ | 5’- TCTTGAAAGACAATCAGGCCATCA -3’  5’- GAATCAGCAGCGACTCCTTTTCC -3’ | 233 |
| IL-4 | 5’-CAAACGTCCTCACAGCAACG-3’  5’-CTTGGACTCATTCATGGTGC-3’ | 203 |
| IL-10 | 5’- GCTCTTACTGACTGGCATGAG -3’  5’- CGCAGCTCTAGGAGCATGTG -3’ | 105 |
| T-bet | 5’-GATCATCACTAAGCAAGGACGGC-3’  5’-AGACCACATCCACAAACATCCTG-3’ | 101 |
| GATA3 | 5’-AGTCCTCATCTCTTCACCTTCC-3’  5’-GGCACTCTTTCTCATCTTGCCT-3’ | 112 |
| STAT-4 | 5’-TGGCAACAATTCTGCTTCAAAAC-3’  5’-GAGGTCCCTGGATAGGCATGT-3’ | 225 |
| STAT-6 | 5’-CTCTGTGGGGCCTAATTTCCA-3’  5’-CATCTGAACCGACCAGGAACT-3’ | 135 |
| CCL2 | 5’-GTCTGTGCTGACCCCAAGAAG-3’  5’-TGGTTCCGATCCAGGTTTTTA-3’ | 62 |
| CCL3 | 5’-ATTCCTGCCACCTGCATAGCT-3’  5’-AGTCCCTCGATGTGGCTACTTG-3’ | 68 |
| CCL5 | 5’-CCTCACCATCATCCTCACTGCA-3’  5’-TCTTCTCTGGGTTGGCACACAC’-3’ | 215 |
| CXCL2 | 5’- CTGAACAAAGGCAAGGCTAA -3’  5’- GCACATCAGGTACGATCCAG -3’ | 125 |
| PTGS2 | 5’- GCAGATGACTGCCCAACTC -3’  5’- CAGGGATGAACTCTCTCCGT -3’ | 104 |
| IL-6 | 5’-ACAACCACGGCCTTCCCTACTT-3’  5’-CACGATTTCCCAGAGAACATGTG-3’ | 129 |
| IL-1β | 5’-TTGACAGTGATGAGAATGACCTG-3’  5’-GCTCTTGTTGATGTGCTGCT-3’ | 137 |
| TNF-α | 5’-CCACCACGCTCTTCTGTCTAC-3’  5’-GAGGGTCTGGGCCATAGAA-3’ | 104 |
| CSF2 | 5’-CTGCGTAATGAGCCAGGAAC-3’  5’-GTTTGTCTTCCGCTGTCCAA-3’ | 126 |

**Supplementary Table 3.** The top 10 hub genes rank in cytoHubba.

| MCC | MNC | Degree | Closeness | Radiality | Stress | EPC |
| --- | --- | --- | --- | --- | --- | --- |
| IL-6 | TNF | TNF | TNF | TNF | TNF | TNF |
| TNF | IL-6 | IL-6 | IL-6 | IL-6 | IL-6 | IL-6 |
| IL-1β | IL-1β | IL-1β | IL-1β | PTPRC | PTPRC | IL-1β |
| IL-10 | IL-10 | IL-10 | PTPRC | IL-1β | IL-1β | PTPRC |
| SYK | PTPRC | PTPRC | IL-10 | SYK | LYN | IL-10 |
| LYN | SYK | LYN | SYK | IL-10 | SYK | LYN |
| VAV1 | LYN | SYK | LYN | CCL2 | HFKB1 | SYK |
| BTK | CCL2 | CCL2 | CCL2 | HFKB1 | FOS | CCL2 |
| LCP2 | PLCG2 | PLCG2 | HFKB1 | LYN | IL-10 | CSF2 |
| ZAP70 | CSF2 | NFKB1 | CSF2 | CSF2 | CCL2 | CCL4 |

**Supplementary Table 4.** Predicted key transcriptional factors (TFs).

| No. | TFs | Description | No. of overlapped genes | *P* value |
| --- | --- | --- | --- | --- |
| 1 | NFKB1 | nuclear factor of kappa light polypeptide gene enhancer in B-cells 1 | 42 | 2.22E-35 |
| 2 | RELA | v-rel reticuloendotheliosis viral oncogene homolog A (avian) | 41 | 3.26E-34 |
| 3 | STAT3 | signal transducer and activator of transcription 3 (acute-phase response factor) | 15 | 2.03E-11 |
| 4 | SPI1 | spleen focus forming virus (SFFV) proviral integration oncogene spi1 | 11 | 3.44E-11 |
| 5 | CEBPA | CCAAT/enhancer binding protein (C/EBP), alpha | 10 | 9.67E-11 |
| 6 | SP1 | Sp1 transcription factor | 24 | 1.25E-10 |
| 7 | JUN | jun proto-oncogene | 14 | 4.68E-10 |
| 8 | STAT1 | signal transducer and activator of transcription 1, 91kDa | 11 | 1.03E-09 |
| 9 | STAT6 | signal transducer and activator of transcription 6, interleukin-4 induced | 7 | 7.29E-08 |
| 10 | NR1I2 | nuclear receptor subfamily 1, group I, member 2 | 6 | 2.18E-07 |

**Supplementary Table 5.** The details of the key targets.

| No. | Gene | Full name | Function |
| --- | --- | --- | --- |
| 1 | PTGS2 | prostaglandin-endoperoxide synthase 2 | Dual cyclooxygenase and peroxidase in the biosynthesis pathway of prostanoids, a class of C20 oxylipins mainly derived from arachidonate, with a particular role in the inflammatory response. |
| 2 | IL-6 | interleukin 6 | Encodes a member of the interleukin family of cytokines that have important functions in immune response, hematopoiesis, inflammation and the acute phase response. |
| 3 | NOS3 | nitric oxide synthase 3, endothelial cell | Produces nitric oxide (NO) which is implicated in vascular smooth muscle relaxation through a cGMP-mediated signal transduction pathway. |
| 4 | SYK | spleen associated tyrosine kinase | Non-receptor tyrosine kinase which regulates several biological processes including innate and adaptive immunity, cell adhesion, osteoclast maturation, platelet activation and vascular development. |
| 5 | VAV1 | vav 1 oncogene | Couples’ tyrosine kinase signals with the activation of the Rho/Rac GTPases, thus leading to cell differentiation and/or proliferation. |
| 6 | LYN | LYN proto-oncogene | Non-receptor tyrosine-protein kinase that transmits signals from cell surface receptors and plays an important role in the regulation of innate and adaptive immune responses, hematopoiesis, responses to growth factors and cytokines, integrin signaling. |
| 7 | NFKB1 | nuclear factor of kappa light polypeptide gene enhancer in B cells 1, p105 | NF-kappa-B is a pleiotropic transcription factor present in almost all cell types and is the endpoint of a series of signal transduction events that are initiated by a vast array of stimuli related to many biological processes such as inflammation, immunity, differentiation, cell growth, tumorigenesis and apoptosis. |
| 8 | TLR8 | toll-like receptor 8 | Endosomal receptor that plays a key role in innate and adaptive immunity. Controls host immune response against pathogens through recognition of RNA degradation products specific to microorganisms that are initially processed by RNASET2. |
| 9 | ZAP70 | zeta-chain (TCR) associated protein kinase | Tyrosine kinase that plays an essential role in regulation of the adaptive immune response. Regulates motility, adhesion and cytokine expression of mature T-cells, as well as thymocyte development. Contributes also to the development and activation of primary B-lymphocytes. |
| 10 | TNF | tumor necrosis factor | This gene encodes a multifunctional proinflammatory cytokine that belongs to the tumor necrosis factor (TNF) superfamily. It plays an important role in the innate immune response as well as regulating homeostasis but is also implicated in diseases of chronic inflammation. |
